# Models of Palliative Care Delivery for Individuals with Cystic Fibrosis: Cystic Fibrosis Foundation Evidence-Informed Consensus Guidelines

**DOI:** 10.1089/jpm.2020.0311

**Published:** 2020-12-17

**Authors:** Dio Kavalieratos, Anna M. Georgiopoulos, Lara Dhingra, Melissa J. Basile, Elliot Rabinowitz, Sarah E. Hempstead, Albert Faro, Elisabeth P. Dellon

**Affiliations:** ^1^ Division of Palliative Medicine, Department of Family and Preventive Medicine, Emory University, Atlanta, Georgia, USA.; ^2^ Department of Psychiatry, Massachusetts General Hospital, Boston, Massachusetts, USA.; ^3^ MJHS Institute for Innovation in Palliative Care, New York, New York, USA.; ^4^ Department of Family and Social Medicine, Albert Einstein College of Medicine, Bronx, New York, USA.; ^5^ Department of Medicine, Northwell Health, Manhasset, New York, USA.; ^6^ Boston Children's Hospital, Boston, Massachusetts, USA.; ^7^ Cystic Fibrosis Foundation, Bethesda, Maryland, USA.; ^8^ Department of Pediatrics, University of North Carolina, Chapel Hill, North Carolina, USA.

**Keywords:** clinical guidelines, cystic fibrosis, healthcare delivery, palliative care, patient-reported outcomes

## Abstract

Cystic fibrosis (CF) affects more than 70,000 individuals and their families worldwide. Although outcomes for individuals with CF continue to improve, it remains a life-limiting condition with no cure. Individuals with CF manage extensive symptom and treatment burdens and face complex medical decisions throughout the illness course. Although palliative care has been shown to reduce suffering by alleviating illness-related burdens for people with serious illness and their families, little is known regarding the components and structure of various delivery models of palliative care needed to improve outcomes for people affected by CF. The Cystic Fibrosis Foundation (CFF) assembled an expert panel of clinicians, researchers, individuals with CF, and family caregivers, to develop consensus recommendations for models of best practices for palliative care in CF. Eleven statements were developed based on a systematic literature review and expert opinion, and address primary palliative care, specialty palliative care, and screening for palliative needs. These recommendations are intended to comprehensively address palliative care needs and improve quality of life for individuals with CF at all stages of illness and development, and their caregivers.

## Introduction

Cystic fibrosis (CF) is a life-limiting genetic disorder affecting more than 70,000 individuals and their families worldwide. Although CF is progressive and ultimately fatal, therapeutic advances have dramatically enhanced life expectancy, with median predicted survival for individuals with CF in the United States increasing from the late 20s in 1986 to 44 years in 2018.^1^ Yet, the multifactorial burdens of the disease continue to profoundly affect the lives of individuals with CF and their families.^2^ CF-related suffering encompasses physical symptoms (e.g., pain, dyspnea, fatigue), emotional distress (e.g., anxiety, depression), impaired function, social isolation, role limitations, treatment-related burden, and existential distress.^2,3^

Palliative care is the overarching approach to care focused on relieving suffering and improving quality of life (QoL) for individuals living with serious illness and their caregivers, from the time of diagnosis forward.^4^ It is a clinical specialty practiced by trained clinicians (i.e., specialty palliative care [SPC]), and may also be delivered by primary providers, including CF care teams (i.e., “primary” palliative care [PPC]).^5^ Although recommended for individuals with lung disease,^6^ integration of palliative care within CF care is rare. While potential explanations for this are varied, formative data we collected from individuals with CF, caregivers, and clinicians suggest that idiosyncratic aspects of the disease render palliative care in CF to be different than in other conditions (e.g., the lifelong nature of the disease, the role of parents/guardians in care and decision making, social isolation due to infection control).^7–10^

As a foundational step toward the creation of guidelines for palliative care in CF, a consensus definition of palliative care in CF was developed by key stakeholders, including individuals with CF, caregivers, CF care team members, palliative care clinicians, and researchers: “Palliative care focuses on reducing physical and emotional symptoms and improving quality of life for people with CF throughout their lives. Palliative care occurs alongside usual treatments and is individualized according to the unique goals, hopes and values of each person with CF.”^11^ As such, this guideline aims to assist clinicians involved in the care of individuals with CF to recognize and adopt tangible practices to address sources of distress among individuals with CF and their caregivers.

## Methods

The Cystic Fibrosis Foundation (CFF) assembled a 22-member committee, led by Drs. Dellon and Kavalieratos, of adults with CF, caregivers, clinicians, researchers, and administrators with expertise in palliative care and/or CF (hereinafter, committee). The committee was convened in July 2018 and was divided into three workgroups: (1) models of palliative care delivery, (2) palliative care skills and training, and 3) screening and assessment of palliative needs. Each workgroup collaborated on 12 anticipated recommendation statements guided by existing frameworks and recommendations in other conditions^6,12–14^ to guide development of PICO-format (Population, Intervention, Comparator, Outcome) questions and a subsequent comprehensive literature review (see Supplementary Appendix A1 for initial PICO questions).

A health sciences librarian (M.K-F.) operationalized each workgroup's PICO questions into a search strategy encompassing MEDLINE, EMBASE, CINAHL, and Cochrane CENTRAL databases (Supplementary Appendix A1). The search identified English-language articles from database inception to March 1, 2018. The screening and assessment workgroup augmented this master search using a recently conducted systematic review of palliative assessment tools^15^ along with recommendations of subject-expert committee members (see Supplementary Appendix A1 for methodology).

Each reference was independently reviewed by two committee members, and, as necessary, adjudicated by one of the two committee leaders. DistillerSR software (Evidence Partners, Ottawa, Canada) was used for article screening and customized, structured forms were developed in a REDCap database to facilitate data extraction from articles.^16^

In January 2019, the committee met in person to finalize the recommendation statements, setting an *a priori* 80% minimum threshold for approval of each statement. Each workgroup presented their revised recommendation statements, including key evidence and expert opinion supporting their recommendations. Committee members not physically present submitted their votes through email. Several statements were subsequently consolidated or reworded; these changes were reviewed by all committee members through email and revoted at the 80% acceptance threshold.

In August 2019, a draft of this article was distributed for a two-week public comment period. Feedback was collected through an internet-based survey, and comments were reviewed by the committee, revising the article as appropriate. Relevant citations published after completion of our initial literature search were added at the discretion of the committee.

## Results

Our searches yielded 8465 references. After removal of duplicates, the committee screened 8298 references; of those, 340 were selected for full-text review (Supplementary Appendix A1), and a final pool of 116 articles were deemed to be potentially relevant to the PICO questions. Ultimately, the committee produced 11 recommendation statements (Table 1) organized in three categories: (1) primary palliative care (PPC); (2) specialty palliative care (SPC); and (3) screening and assessment of palliative care needs. All of the proposed recommendation statements passed the 80% threshold for acceptance.

**Table 1.** Summary of Recommendation Statements

Recommendation statements			
Topic	Number	Recommendation	Percent agreement among committee members
Primary palliative care	1	The CF Foundation recommends that CF care teams deliver PPC as part of usual CF care, at the time of diagnosis and throughout the disease course.	100
	2	The CF Foundation recommends that CF care team members receive PPC training relevant to their discipline and employ these skills within their scope of practice.	100
	3	The CF Foundation recommends that CF and transplant care teams engage individuals with CF and their caregivers in goals-of-care discussions and advance care planning across the lifespan to align the care received with their values, preferences, and priorities.	100
	4	The CF Foundation recommends that CF and, if applicable, transplant care teams take a collaborative approach in offering comprehensive, timely, and compassionate end-of-life care, including (but not limited to) hospice services, to individuals with CF and provide clinical expertise and support through the end of life.	100
	5	The CF Foundation recommends that CF care teams identify and address caregivers' concerns, and provide support and resources for caregivers outside the CF care team when appropriate, from diagnosis through bereavement.	95
SPC consultation	6	The CF Foundation recommends that CF and, if applicable, transplant care teams consult SPC clinicians and other specialists to address palliative care needs beyond their expertise, facilitating seamless communication pathways between and among teams.	100
	7	The CF Foundation recommends SPC consultation when an individual with CF is considering or declines transplantation.	100
	8	The CF Foundation recommends that CF care teams partner with specialists who are consulted to assist with palliative care needs to facilitate the specialists' understanding of CF care and the unique needs of individuals with CF.	100^a^
Screening and assessment of palliative care needs	9	For individuals with CF ages 12- to adulthood, the CF Foundation recommends using the IPOS, annually and at disease milestones (e.g., changes in disease severity, functional decline), for screening and clinical assessment of unmet palliative care needs.	100
	10	For children with CF under age 12 years, the CF Foundation recommends using the IPOS to guide conversations with children and caregivers, annually and at disease milestones (e.g., changes in disease severity, functional decline), to identify unmet palliative care needs.	100
	11	For caregivers of individuals with CF of all ages, the CF Foundation recommends offering screening to at least one primary caregiver annually and when disease milestones (e.g., changes in disease severity, functional decline) trigger repeated screening, using the BASC.	100

^a^ One committee member abstained.

BASC, Brief Assessment Scale for Caregivers; CF, cystic fibrosis; IPOS, Integrated Palliative Care Outcome Scale; PPC, primary palliative care; SPC, specialty palliative care.

### Recommendation statements

#### Primary palliative care

##### *Recommendation 1:* The CFF recommends that CF care teams deliver primary palliative care as part of usual CF care, at the time of diagnosis, and throughout the disease course

**FIG. 1.**
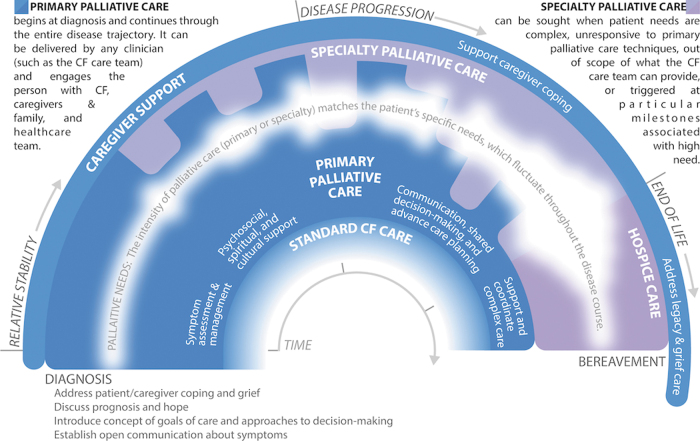
Integrating primary and specialty palliative care to address palliative needs throughout the CF experience. Palliative care is delivered continuously throughout the illness experience, from diagnosis through bereavement. The intensity of palliative care should match the patient and family's palliative needs, including but not limited to: assessment and management of physical and emotional symptoms; existential and spiritual suffering; provision of information regarding prognosis and treatment options, elicitation of goals of care, and enactment of advance care planning; and support during transplant evaluation and post-transplantation. Palliative care is ideally a partnership, whereby CF care team members provide continuous first-line monitoring and support for palliative needs (“primary palliative care”), while palliative care specialists support the CF care team with expert management of complex or severe concerns. Palliative care needs are illustrated as the variable white band throughout the center of the figure; the width and opacity of the band indicate the fluctuating severity of palliative needs. Importantly, this figure is a crude depiction of palliative needs, which will vary by patient/family; as such, CF care teams should continuously monitor suffering and needs to customize palliative care for each patient. Furthermore, this illustration does not specifically depict certain milestones, such as the transplantation process, which may also affect palliative needs. Color image is available online.

Attending to suffering and delivering care that is concordant with a patient's goals are central to providing high-quality care to individuals with serious illnesses like CF.^5,6^ Historically, palliative care has been falsely conceptualized as a choice between life-sustaining and comfort-focused treatment.^17^ However, research demonstrates that a longitudinal, palliative approach to care benefits patients and families well before the very end of life.^18^ Furthermore, waiting to provide palliative care until a trigger event such as a major decline in health status reinforces the misconception that palliative care is an unfortunate option of last resort. Instead, palliative care should be delivered throughout the entire course of illness, and its intensity modulated to match the severity of a patient's needs (Fig. 1). Given the strong relationships that exist between CF care teams and individuals with CF and their families, CF care team members are ideally suited to provide continuous first-line monitoring and management of basic palliative care needs (i.e., PPC), whereas palliative care specialists can offer expert management of complex or intractable distress that is beyond the scope or skillset of the CF care team (i.e., SPC). In fact, many tasks already delivered by CF care teams at the time of diagnosis, such as discussing symptoms and expectations for the future, would be considered “primary palliative care” even if not explicitly labeled as such to patients and their families. This recommendation reinforces the importance of PPC as a part of usual CF care and recognizes the capacity of CF care teams to deliver these elements throughout the entire disease trajectory.^19–21^ Ideally, the provision of palliative care, like standard CF care, is a coordinated, patient-driven partnership by an interdisciplinary team of professionals.

##### *Recommendation 2:* The CFF recommends that CF care team members receive PPC training relevant to their discipline and employ these skills within their scope of practice

CF care team members endorse the need for focused training in PPC skills.^8,10,22,23^ Relevant PPC skills include basic pain and symptom management and communication about goals of care, prognosis, and treatment decisions.^5,9,23^ Table 2 outlines examples of how different CF care team members can address the varied palliative care needs of individuals with CF.^24^ Linnemann and colleagues developed a CF-specific palliative care curriculum for potential implementation in CF care centers,^22^ and a toolkit and implementation guide developed by this Committee is available in the United States CFF Resources Library. Rigorously developed and widely accessed palliative care training, even if not CF specific, is also available through a variety of conferences and other trainings (Table 3). Additionally, local palliative care experts can provide education and support to their affiliated CF care teams.^25^

**Table 2.** Primary versus Specialty Palliative Care for Individuals with Cystic Fibrosis and Their Families

Palliative care domain	PPC: Palliative care concerns addressed by CF care team	SPC: Reasons to consider consulting a palliative care specialist
Symptom management	□ Basic management of pain and other physical symptoms □ Basic management of anxiety, depression	□ Assist with managing physical symptoms refractory to PPC interventions □ Assist with managing emotional symptoms refractory to PPC interventions □ Address existential/spiritual distress
Communication and advance care planning	□ Educate about CF as a chronic, progressive condition □ Discuss prognostic uncertainty □ Communicate distressing news related to CF □ Engage patient and caregivers in discussions about goals of care □ Describe options for life-sustaining treatments for respiratory failure □ Identify surrogate decision maker □ Guide documentation of legal, actionable advance directives □ Educate and support around transplantation, including medical indications, processes, and outcomes	□ Address code status, advance directives when there are misunderstandings of options and prognosis □ Navigate discordance among patients, caregivers, and health care providers □ Address concerns about misalignment of goals and treatment decisions □ Address fears about future illness and preferences for communication (e.g., family vs. patient-centered, amount of information desired about illness, and benefits versus burdens of treatment options) □ Act as third party for conversations about transplantation □ Additional exploration of wishes around end-of-life care
Caregiver support	□ Supportive/empathic listening □ Screen for caregiver anxiety and depression □ Identify resources for emotional support □ Address financial needs and identify resources	□ Address needs that exceed expertise of CF care team, particularly if conflict exists □ Address existential/spiritual distress of caregivers □ Explore grief and bereavement needs and assist with community referrals
Care coordination	□ Communicate with other relevant health care providers □ Referral to community resources	□ Communicate with CF care team and other relevant health care providers □ Introduction and revisiting of hospice and community palliative care resources; hospice eligibility review

Adapted with permission from Kavalieratos et al.^18^

**Table 3.** Resources for Palliative Care Training for Cystic Fibrosis Care Team Members

Resource or organization	How to access	Conferences	Videos	Printed materials	Courses
Annual Assembly of Hospice and Palliative Care	www.aahpm.org	✓			
Center to Advance Palliative Care	www.capc.org/training	✓	✓	✓	✓
Cystic Fibrosis Foundation Patient Registry [PortCF] Resources Section	portcf.cff.org			✓	
	These resources are password protected. To access them, contact your PortCF administrator.				
Cystic Fibrosis Foundation Resources Library	my.cff.org			✓	
	The resources are password protected; all care team members are able to access this portal. Individuals not listed as care team members need to request access.				
Courageous Parents Network	https://courageousparentsnetwork.org/		✓	✓	
Education on Palliative and End-of-Life Care Project (EPEC)	www.bioethics.northwestern.edu/programs/epec				✓
End-of-Life Nursing Education Consortium (ELNEC)	www.aacnnursing.org/ELNEC			✓	✓
MJHS Institute for Innovation in Palliative Care	https://www.mjhspalliativeinstitute.org/e-learning/		✓	✓	
National Hospice and Palliative Care Organization	www.nhpco.org/education	✓	✓	✓	✓
The Conversation Project	www.theconversationproject.org		✓	✓	
VitalTalk	www.vitaltalk.org		✓	✓	✓

##### *Recommendation 3:* The CFF recommends that CF and transplant care teams engage individuals with CF and their caregivers in goals-of-care discussions and advance care planning across the lifespan to align care with their values, preferences, and priorities

Goals of care represent an individual's unique hopes and preferences for medical care in the context of disease status, values, beliefs, and culture.^14^ Advance care planning (ACP) is a process of considering and documenting wishes for future medical care. ACP is a continuum from elicitation of goals of care through the creation of advance directives, such as legal documents about treatment preferences or surrogate decision makers. ACP is recommended for people with CF and other serious illnesses.^6,14,26,27^ Delaying ACP until the late stages of disease is common in CF ^7,9,28–32^ and other serious illnesses, but early ACP affords improved communication among individuals with CF, caregivers, and health care providers, and alignment of goals of care with treatment decisions.^14,33^ Studies in CF indicate unmet ACP needs and patient willingness to engage in ACP conversations.^7,9,31,34^ Goals of care and ACP should also be addressed throughout the transplant process, from consideration of referral through post-transplant care, as new decisions are faced and goals may be everchanging.^35,36^ Communication about goals of care between CF and transplant teams who comanage patients is essential, as is engaging individuals with CF and caregivers in effective goal-setting discussions. Resources to facilitate ACP conversations are available in the U.S. CFF Resources Library.

Novel, highly effective modulator therapies, such as elexacaftor/tezacaftor/ivacaftor, hold the promise of dramatically extending survival for ∼90% of the CF population.^37^ Yet, the need for early, proactive, and iterative goals-of-care elicitation and ACP remains important as a way to “hope for the best, and prepare for the worst.”^38^ First, without long-term surveillance studies, it is uncertain whether projected increases in survival will ultimately be realized. Second, it is unclear what the physical and psychosocial ramifications are of extending survival for individuals with existing organ damage, along with the burdens of coping with an uncertain prognosis. Third, even with modulator therapy, it is estimated that up to 6% of individuals with CF will still require lung transplantation in the next 20 years.^39^

##### *Recommendation 4:* The CFF recommends that CF and, if applicable, transplant care teams take a collaborative approach in offering comprehensive, timely, and compassionate end-of-life care, including (but not limited to) hospice services, to individuals with CF and provide clinical expertise and support through the end of life

As individuals with CF approach the final stages of their lives, the CF care team remains critically important in providing comfort-focused care to alleviate suffering for patients and families. Yet, research suggests that current practices regarding end-of-life care in CF are suboptimal. Caregivers of individuals with CF commonly report distressing symptoms, often with little support for or expectations of symptom control.^40^ Hospice is a health delivery system and entitlement (in the United States) that provides specialist-level palliative care for eligible individuals whose estimated prognosis is six months or less and elect to forgo life-sustaining treatments. It provides access to enhanced symptom management and supportive services, such as physical and occupational therapy, social work, pastoral care, and bereavement support.^4^ In a retrospective analysis of 248 people with CF who died between 2011 and 2013, only 32% received hospice; hospice enrollment was associated with a lower likelihood of death in intensive care and a higher rate of prior ACP.^30^ As evidence shows that hospice improves clinical outcomes at the end of life, including QoL,^41^ symptom control,^41^ and care satisfaction,^41^ and also reduces avoidable health care expenses,^42^ hospice discussions between CF care teams and patients and families should be proactive. Yet anecdotally, appropriate hospice referral is commonly impeded in CF, particularly because of the cost of CF medications. Given that hospice reimbursement is structured on a *per diem* basis that does not account for the complexity or cost of a specific patient's treatment needs, many hospice organizations have policies that limit access for patients with costly treatments.^43^ In turn, people with CF and their care teams often are forced to choose between maintenance therapies that provide symptom relief versus the comprehensive expert end-of-life management that hospice provides.

Recognizing that barriers may exist to accessing hospice services, CF care teams should consider early engagement of experts in collaborative management of end-of-life concerns, including palliative care specialists, chaplains, and social workers.^17^ Even following the individual's referral to hospice, CF care teams should remain an integral component of the patient and family's end-of-life experience. In addition to mitigating patient and family fears of abandonment,^44^ CF care teams provide critical expertise regarding nuanced disease processes and management to other clinicians.

##### *Recommendation 5:* The CFF recommends that CF care teams identify and address caregivers' concerns and provide support and resources for caregivers outside the CF care team when appropriate, from diagnosis through bereavement

Caregivers are central in the care of individuals with CF throughout the lifespan.^45^ Caregiving roles change over time, with both the degree and types of physical and emotional support changing depending on the health status of individuals with CF.^46^ Caregiver burdens and needs may be amplified with disease progression, and as the CF population ages with improved outcomes, caregiving duties are shifting from parents to partners, and even to children of individuals with CF.

Caregiving brings countless rewards but also substantial emotional and financial tolls; thus, attention to caregiver needs is critical.^13,47^ Caregiver concerns may include worries about the individual with CF, feeling overwhelmed or depressed, distress around medical decision making, inability to attend to one's own needs and other responsibilities, strained relationships, and the emotional and existential impact of caregiving.^48^ While the CF care team is able to provide education and emotional support, examples of external resources that may benefit caregivers include caregiver support groups, mental health services, community resources for nursing and/or respite care, and hospice for bereavement care.

#### Specialty palliative care consultation

##### *Recommendation 6:* The CFF recommends that CF and, if applicable, transplant care teams consult SPC clinicians and other specialists to address palliative care needs beyond their expertise, facilitating seamless communication pathways between and among teams

Nonhospice palliative care is indicated by patient need, not prognosis; therefore, CF care teams should consistently and proactively assess palliative care needs throughout the entire disease course to determine whether a PPC approach is sufficient or if SPC is necessary.^5,6^ Recognizing when and how to optimally engage SPC clinicians and other specialists underlies the ability to use these resources effectively and efficiently.^5,17,49^ Figure 1 and Table 2 delineate common palliative care needs faced by individuals with CF and their families and offer suggestions for when the involvement of specialists (palliative care or other) may be beneficial. Specific indications for when SPC may be beneficial include, but are not limited to: the presence of complex or intractable symptoms; existential or spiritual distress; family conflict regarding goals of care; during the transplant evaluation process; and when illness is advanced or death is perceived to be soon.^50,51^ Furthermore, the involvement of palliative care specialists may enhance the confidence and competence of CF care teams to provide PPC.^25^

##### *Recommendation 7:* The CFF recommends SPC consultation when an individual with CF is considering or declines transplantation

Although SPC should be considered at any point in the disease course as indicated by unmet need, the CFF recommends involvement of SPC when transplantation becomes a viable treatment option. Currently, SPC is rarely involved in the care of patients undergoing the lung transplantation process,^35,52^ and when it is, patients often present with greater illness burden and disability compared with cancer patients also referred for SPC.^50^ Well-documented barriers impede the use of SPC, including patient and clinician difficulties with acceptance of prognosis, clinician fears of demoralizing patients, fears regarding abandonment, unrealistic patient and family expectations regarding survival, and family conflict regarding goals of care.^44^ Addressing the last two barriers are among SPC's strongest contributions to clinical care.^4^ Indeed, the benefits of specialty palliative care in transplantation may principally address the benefits versus burdens of this high-risk, complex treatment option. A 2009 qualitative study of surviving caregivers of individuals with CF who had received lung transplants revealed various informational deficits regarding risks and alternatives to transplant; specifically, 46% of caregivers expressed that the patient did not understand that declining transplant was an option.^53^ Beyond providing valuable support to families and CF care teams in the decision-making process,^29^ SPC services may be helpful in improving or stabilizing the complex symptom burden to be expected at the point of transplant decisions.^50^ As such, regardless of the decision to undergo or decline transplantation, SPC can assist CF care teams in optimizing QoL for individuals with CF and their families at this difficult milestone.

##### *Recommendation 8:* The CFF recommends that CF care teams partner with specialists who are consulted to assist with palliative care needs to facilitate the specialists' understanding of CF care and the unique needs of individuals with CF

Embedded models of SPC within CF care teams can address illness burden and enhance QoL by integrating specialist-level palliative care expertise within usual CF care.^54–57^ For example, a recent pilot randomized clinical trial of embedded SPC for adults with CF showed high feasibility and acceptability, and is currently being tested for effectiveness in a phase III trial.^55^ However, institutional resources often limit access, such that most palliative care specialists serve patients with varied medical conditions in inpatient settings, while outpatient palliative care programs are almost exclusively focused on oncology and may devote little time to CF care; these barriers are in addition to shortages in the SPC workforce.^49^ Given the unique needs of individuals with CF, the growing recognition that palliative care services should be adapted to address these needs,^31^ and a paucity of CF-specific educational resources for palliative care specialists,^58^ CF care teams must support palliative care specialists in understanding CF-specific concerns.^54,59^ Additionally, open communication and thoughtful partnerships will facilitate role delineation, which is important to building trust and reducing duplication of services.

#### Screening and assessment of palliative care needs

##### *Recommendation 9:* For individuals with CF, ages 12 and older, the CFF recommends using the Integrated Palliative Care Outcome Scale, annually and at disease milestones (e.g., changes in disease severity, functional decline), for screening and clinical assessment of unmet palliative care needs

**FIG. 2.**
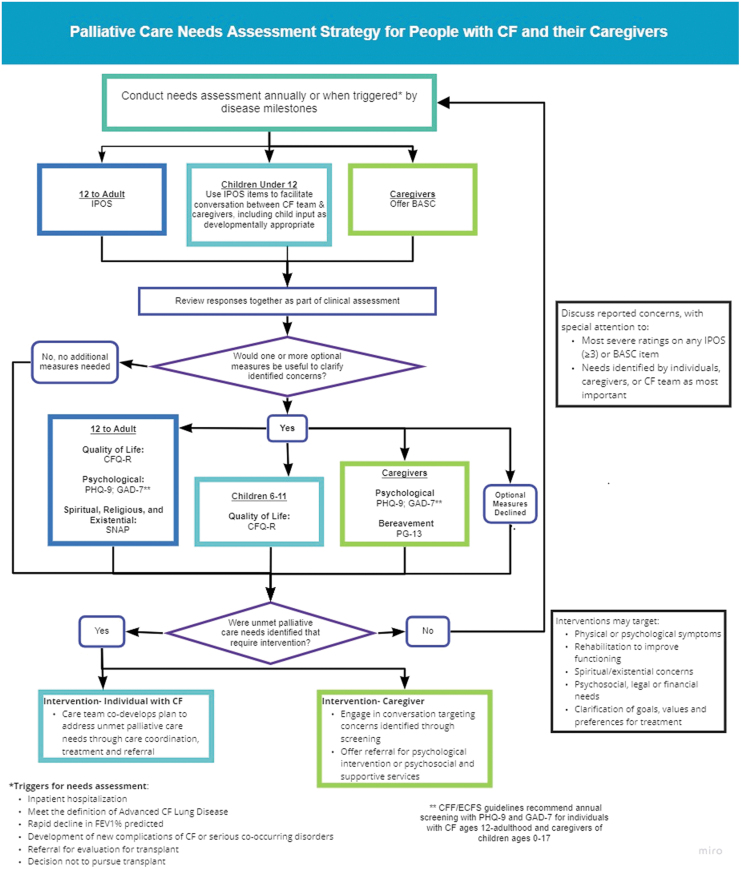
Palliative care needs assessment strategy for people with CF and their caregivers. Color image is available online.

The CFF recommends that CF centers screen individuals for unmet palliative care needs annually throughout the illness course (Table 4; Fig. 2). Given the unpredictable and varied course of CF, in some individuals annual screening will be insufficient to identify palliative care needs and promote timely intervention. Screening triggered by changes in disease severity, functional decline, or after major events like hospitalization, transplantation, or newly emergent comorbidities, prompts CF teams to rescreen throughout the illness trajectory as new needs arise.

**Table 4.** Recommended and Optional Measures for Screening of Palliative Care Needs

Measures recommended for annual and triggered screening					
Measure	Target population			Palliative care domains assessed^61^	How to interpret elevated scores
	Individuals with CF ages <12 years	Individuals with CF ages 12+	Family caregivers		
IPOS^54^	Use items to facilitate communication between CF team and caregivers, including child input as developmentally appropriate	Completed by self-report	—	□ Structure and processes of care □ Physical aspects of care □ Psychological aspects of care □ Social aspects of care □ Spiritual, religious, and existential aspects of care	Discuss reported concerns Special attention to most severe ratings: ≥3 on any item
BASC^42,67,68^	—	—	✓	□ Social: Caregiver burden and distress	Discuss reported concerns Special attention to most severe rating on any item
*Optional measures endorsed for additional assessment*					
*Measure*	*Target population*			*Palliative care domains assessed*	*How to interpret elevated scores*
	*Individuals with CF ages <12 years*	*Individuals with CF ages 12+*	*Family caregivers*		
CFQ-R^55,56^	Any age-appropriate CFQ-R Subscales: □ Physical functioning □ Emotional functioning □ Role perception □ Social perception □ Health perception □ Body image □ Eating disturbance □ Treatment burden □ Vitality □ Respiratory symptoms □ Digestive symptoms □ Weight		—	□ Physical □ Psychological	Discuss moderate to severe ratings on any item *or* Calculate standardized subscale T-scores
PHQ-9^58^	—	✓	✓	□ Psychological: Depression	Mild: 5–9 Moderate: 10–14 Severe: 15+
GAD-7^59^	—	✓	✓	□ Psychological: *Anxiety*	
SNAP^61^	—	✓	—	□ Spiritual, religious, and existential	Very much or somewhat on any item
PG-13^69^	—	—	✓	□ Care at the end of life: Bereavement	Distress, duration, symptoms, impairment

The CFQ-R is available in multiple versions, depending on respondent and respondent age: Parent/Infant-Preschool CFQ-R for Children Ages 0 to 5; Preschool CFQ-R for Children Ages 3 to 6; CFQ-R for Children Ages 6 to 11, Interviewer or Parent Version; CFQ-R for Children Ages 12 and 13, Self-report or Parent Version; CFQ-R for Adolescents and Adults 14 Years Old and Older.

BASC, Brief Assessment Scale for Caregivers; CFQ-R, Cystic Fibrosis Questionnaire–Revised; GAD-7, Generalized Anxiety Disorder 7-item Scale; IPOS, Integrated Palliative Care Outcome Scale; PG-13, Prolonged Grief Questionnaire; PHQ-9, Patient Health Questionnaire-9; SNAP, Spiritual Needs Assessment for Patients.

The Integrated Palliative Care Outcome Scale (IPOS) is a brief, well-validated multidimensional measure that is widely used in seriously ill adult populations, including outpatients.^60^ It identifies clinical needs for targeted palliative interventions in domains that are highly relevant yet not expressly captured in available CF-specific tools, such as pain, communication, spiritual, and financial concerns. It can be administered through self-report in adults and adolescents. The CF team should frame screening in a manner sensitive to personal context and priorities. Before offering screening measures, a trusted CF team member should convey: (1) the rationale for universal screening to facilitate early intervention for symptoms and problems affecting QoL; and (2) the process by which the team member and individual with CF (along with caregiver(s) as appropriate to developmental stage and preference), will review responses together to cocreate an action plan to address concerns.

When newly identified palliative care needs are detected following screening, additional measures can facilitate clinical assessment (Table 4). The Cystic Fibrosis Questionnaire—Revised (CFQ-R) is a widely used, CF-specific health-related QoL measure that is well validated in nationally representative samples.^61,62^ CFQ-R subscales may be selected for exploration and repeated assessment of physical symptoms, treatment burden, and self-perceived health and psychosocial status. Of note, the CFQ-R Physical Functioning subscale assesses functional impairment and is predictive of disease-related mortality.^63^ The Patient Health Questionnaire-9^64^ (PHQ-9) and Generalized Anxiety Disorder 7-item Scale^65^ (GAD-7), presently used in most CF centers as per the CFF/European Cystic Fibrosis Society (ECFS) Consensus Statements for Screening and Treating Depression and Anxiety, can be used to assess the severity of depression and anxiety in adolescents and adults with CF.^66^ The Spiritual Needs Assessment for Patients (SNAP) is a practical measure that assesses individuals' desire for help with specific unmet psychosocial, spiritual/existential, and religious needs.^67^ The SNAP might be offered, for example, to individuals who respond “occasionally” or “not at all” to IPOS item 6, “Have you felt at peace?” or who raise concerns about spiritual distress during discussion.

A CF clinician should offer clinical assessment to further evaluate palliative care needs and codevelop a plan of care, with special attention to severe ratings on any of the IPOS items. Clinical assessment may suggest the need for additional treatment, care coordination, or referral for services to address unmet palliative care needs concurrent with usual care. Interventions may target physical or psychological symptoms; rehabilitation to improve functioning; spiritual/existential concerns; psychosocial, legal or financial needs; and clarification of goals, values, and preferences for treatment.

It is important to note that several of the screening tools included in these recommendations, like the IPOS, have yet to be validated in CF populations, and the extent to which this measure is both acceptable and clinically relevant for CF patients with varying levels of disease severity and demographics is an area for additional investigation. For example, some symptoms contained on the measure, such as sore/dry mouth and poor mobility, are likely to have a relatively low prevalence rate in individuals with CF. Similarly, the IPOS does not assess palliative care topics particularly relevant to CF, such as treatment burden, body image, social or school functioning, resilience, or wellness. However, the IPOS contains an open-ended question concerning the individual's main challenges and allows space for additional symptoms to be added, if desired. Furthermore, the items identify clinical needs for targeted CF interventions that are likely to be important contributors to illness burden in this population and are distinct from those elicited by available CF-specific tools: pain, coordination of health care, financial constraints, communication problems, and spiritual concerns.

##### *Recommendation 10:* For children with CF under 12 years of age, the CFF recommends using the IPOS to guide conversations with children and caregivers annually and at disease milestones (e.g., changes in disease severity, functional decline) to identify unmet palliative care needs

Clinical experience and report of individuals with CF and their caregivers suggest that even the youngest individuals with CF may have unmet palliative care needs.^40,68,69^ Yet we did not identify any brief, validated instruments to comprehensively screen for palliative care needs frequently occurring in children with CF. Although the IPOS has face validity for adolescents, evidence is lacking for its use by younger children (either by self- or proxy-reporting). However, a CF care team member may use items from IPOS to facilitate communication about a younger child's symptoms and the family's experience of living with CF, incorporating input from the child as appropriate to developmental stage. This may involve using simpler language and open-ended questions that build on the natural flow of the conversation. For example, instead of asking a child to rate how “poor mobility” has affected her during the previous week on a scale from 0 to 4 (IPOS item 2), the CF team member might ask, “You said you have been tired and coughing a lot this week. How hard is it for your body to move around?”

For centers with broader screening capabilities, age-appropriate CFQ-R subscales may be selected for exploration and repeated assessment of physical symptoms, treatment burden, and self-perceived health and psychosocial status^70–72^; the CFQ-R Emotional Functioning subscale, for example, can elicit psychological symptoms in children ages 6–11 who are too young to complete the PHQ-9 and GAD-7. As with older children and adults with CF, clinical assessment may suggest the need for additional treatment, care coordination, or referral for services to address unmet palliative care needs concurrent with usual CF care.

##### *Recommendation 11:* For caregivers of individuals with CF of all ages, the CFF recommends offering screening to at least one primary caregiver annually and when disease milestones (e.g., changes in disease severity, functional decline) trigger repeated screening, using the Brief Assessment Scale for Caregivers

Validated among caregivers of individuals receiving palliative care, the Brief Assessment Scale for Caregivers (BASC) has been studied in caregivers of adults with CF.^48,73,74^ The BASC identifies targets for clinical follow-up and management of caregiver burden.^48^ In addition, the BASC includes items related to family functioning that may identify concerns for which referral to supportive services may be beneficial. Caregiver screening is meant as a springboard for a nonjudgmental, collaborative discussion of caregiver concerns, with special attention to any BASC item with the most severe rating.

CFF/ECFS Consensus Statements for Screening and Treating Depression and Anxiety recommend offering annual screening with the PHQ-9 and GAD-7 for depression and anxiety, respectively, to parent caregivers of individuals with CF from birth to age 17.^66^ After screening using BASC, if further evaluation is needed, the PHQ-9 and GAD-7 are also suggested as an option for caregivers (parents, partners/spouses, or siblings) of *adults* with CF, consistent with best practices to address caregiver needs across the lifespan. Triggered screening following the death of an individual with CF of any age may also enable CF Centers to support bereaved caregivers. Centers developing bereavement programs may consider offering the Prolonged Grief Questionnaire (PG-13), which has predictive validity for clinical criteria of persistent complex bereavement disorder.^75^

## Conclusions

Addressing sources of suffering among individuals with CF and their caregivers while concurrently focusing on improving outcomes is likely to enhance QoL and ease the burden of living with CF. The multidisciplinary models of CF care, transplant care, and SPC lend themselves naturally to collaboratively provide comprehensive and compassionate attention to the unique palliative care needs of individuals with CF and their caregivers.

These recommendations are intended to assist clinicians in identifying and addressing palliative care needs in individuals with CF and their caregivers across the continuum of illness. Additionally, they attend to the educational needs of CF care team members and palliative care clinicians, encouraging partnerships among all stakeholders. Implementation of these guidelines will vary among centers based on resources, but it is anticipated that most CF care programs will be able to systematically assess for needs and offer guidance and support around each recommendation, while palliative care specialists will support CF clinicians to develop skills in PPC, as well as increasing rates of consultation.

## Supplementary Material

Supplemental data

## Supplementary Material

Supplementary Appendix A1
