# Supplementary material for: Models of Palliative Care Delivery for Individuals with Cystic Fibrosis: Cystic Fibrosis Foundation Evidence-Informed Consensus Guidelines
Source: J Palliat Med. 2020 Dec 17;24(1):18–30. doi: 10.1089/jpm.2020.0311 (PMC7757696; doi:10.1089/jpm.2020.0311)
Supplement: Supplemental data [file Supp_Appendix1.docx]

**SUPPLEMENTARY APPENDIX A1.**

Table of Contents

1. PICO questions designed to guide literature search
2. PubMed/Medline search strategy
3. Flow diagram of studies identified and reviewed to support the development of recommendation statements based on the Preferred Reporting of Items for Systematic Review and Meta-Analysis protocol
4. Flow diagram of palliative care needs assessment tools identified by Screening and Assessment Workgroup (Workgroup 3) based on the Preferred Reporting of Items for Systematic Review and Meta-Analysis protocol
5. Screening and Assessment Workgroup (Workgroup 3) review strategy
6. **PICO Questions Used to Guide Literature Search**

| **Workgroup** | **Inclusion criteria** | **Exclusion criteria** |
| --- | --- | --- |
| **Delivery models of palliative care** | **1.1. What is the evidence supporting models of palliative care service delivery for patients with life-limiting illnesses?**  *Population:* Defined as advanced illnesses) that are similar to cystic fibrosis, in terms of disease trajectory (i.e., a fluctuating course characterized by acute exacerbations with varied periods of stability), symptom burden, and illness burden (such as sickle cell disease; muscular dystrophy; chronic kidney disease; pulmonary hypertension; congenital heart disease; selected chronic obstructive pulmonary disease; HIV; non-metastatic cancer; and rheumatologic disorders).  *Intervention:* Self-described as “palliative care” and/or comprises at least two domains of palliative care, as defined by the National Consensus Project for Quality Palliative Care, or includes advance care planning/goals of care discussions  *Comparators*: Usual care; enhanced usual care; attention control  *Outcomes:* Study reports on at least one or more of the following specified review outcomes: patient quality of life; spiritual wellbeing; physical and psychological symptom burden; symptom distress; illness burden; mood; caregiver burden; advance care planning; satisfaction with care; health service utilization; costs-of-care; length of enrollment in palliative care program/duration of services; length of survival; site of death | Life-limiting illnesses not similar to cystic fibrosis, including neurodegenerative disease such as dementia; metastatic cancer; and other diseases that present acutely in the ICU or hospital setting    Studies not published in English.  *Intervention*: Pharmacologically-oriented or drug-exclusive interventions (e.g., morphine for dyspnea) or study does not otherwise meet this review’s definition of “palliative care” based on National Consensus Project for Quality Palliative Care  *Intervention*: Family caregiver is the exclusive or primary target of intervention; Bereavement care interventions. |
|  | **1.2. What is the evidence supporting models of palliative care service delivery for patients with cystic fibrosis?**  *Population:* Cystic fibrosis (adult and pediatric)  *Intervention:* Self-described as “palliative care” and/or targets either one or both of the following: 1) at least two domains of palliative care, as defined by the National Consensus Project for Quality Palliative Care, including advance care planning/goals of care discussions or 2) at least two *other* domains of illness burden: medical burden; symptom burden; symptom distress; family/caregiver burden; treatment burden/complexity of care  *Study design*: All study designs including quality improvement studies  *Comparators*: All  *Outcomes:* Study reports on at least one or more of the following specified review outcomes: patient quality of life; spiritual wellbeing; physical and psychological symptom burden; symptom distress; illness burden; mood; caregiver burden; advance care planning; satisfaction with care; health service utilization; costs-of-care; length of enrollment in palliative care program/duration of services; site of death | Studies targeting patients without cystic fibrosis  Other exclusion criteria same as 1.1. |
| **Palliative care skills** | **2.1. What are the educational needs and skill set of primary clinicians (i.e., non-palliative care specialists) caring for patients with serious illness?**  *Population:* Primary clinicians (primary care or specialist, but not palliative care specialists) caring for patients (CF and other analogous conditions; see PICO 1.1 for definition)  *Intervention:* Education in 1) basic physical, emotional, and spiritual care management, 2) communication, goals of care, decision making, advance care planning, and/or 3) end of life care, bereavement  *Study design*: all, including qualitative designs and opinion pieces.  *Comparison*: N/A  *Outcomes*: 1) Clinician self-reported confidence in management of a) basic physical, emotional, and spiritual care management, b) communication, goals of care, decision making, advance care planning, and/or c) end of life care, bereavement; 2) documented assessment and/or management of a) basic physical, emotional, and spiritual care management, b) communication, goals of care, decision making, advance care planning, and/or c) end of life care, bereavement; 3) patient-reported outcomes regarding a) physical, emotional, and spiritual care management, b) communication, goals of care, decision making, advance care planning, and/or c) end of life care, bereavement |  |
| **Palliative care skills** | **2.2. What are the educational needs and skill set of palliative care clinicians caring for people with serious illness?**  *Population:* Palliative care clinicians caring for seriously ill patients (CF and other analogous conditions; see 1.1. for definition)  *Intervention:* Education in 1) palliative care of people with CF and 2) palliative care of people with serious illnesses other than CF  *Study design*: all  *Comparison*: N/A  *Outcomes*: 1) Self-reported confidence in providing palliative care to a) patients with CF and/or b) patients with other serious illness; 2) patient-reported outcomes of palliative care including a) patients with CF and b) patients with other serious illness |  |
| **Palliative needs assessment** | **3.1. What tools exist to assess whether the palliative care needs of people with CF (or analogous diseases) or their caregivers are met in various domains?**  *Population:* 1) Individuals with CF of all ages and stages of disease including a) at diagnosis, b) under age 18, c) 18 years and older, d) pre- or post-lung or liver transplant, e) end of life, and 2) caregivers of individuals with CF of all ages and stages of disease including a) at diagnosis, b) under age 18, c) 18 years and older, d) pre- or post-lung or liver transplant, e) end of life; and, 3) patients with analogous diseases, as defined in 1.1.  *Intervention:* Studies that administer or validate standardized assessments related to needs for services in one or more palliative care domains, including multidimensional tools (e.g. HRQoL): 1) PRO tools, 2) caregiver self-report tools, 3) other standardized assessment tools including structured or semi-structured interviews  Palliative care domains: physical symptoms; psychological symptoms and/or conditions; quality of life; advance care planning; healthcare decision-making; spirituality; caregiver quality of life; caregiver psychological symptoms and/or conditions; caregiver burden/stress.  *Study design*: all  *Comparison*: N/A  *Outcomes*: N/A |  |

**2. PubMed/Medline Search Strategy**

The search strategy developed for PubMed/Medline is offered below. Search strategies for other bibliographic databases used are available upon request.

(((((((((((((((("Cystic Fibrosis"[Mesh] OR Cystic Fibrosis Foundation[Corporate Author] OR ECORN-CF Study Group[Corporate Author] OR ECORN-CF[All Fields] OR "European cystic fibrosis"[All Fields] OR “European Cystic Fibrosis Society”[Corporate Author] OR ECFS Board[Corporate Author] OR “European Cystic Fibrosis Society (ECFS) and the ECFS Clinical Trial Network”[Corporate Author] OR “European Cystic Fibrosis Society Clinical Trial Network Standardisation Committee”[Corporate Author] OR “International Committee on Mental Health”[Corporate Author] OR “ERS/ECFS Task Force on Provision of Care for Adults with Cystic Fibrosis in Europe”[Corporate Author] OR "Journal of cystic fibrosis : official journal of the European Cystic Fibrosis Society"[Jour] OR “US Cystic Fibrosis Foundation and European Cystic Fibrosis Society”[Corporate Author] OR "Cystic Fibrosis"[All Fields] OR “cystic lung disease”[tw] OR "CF Foundation"[tw] OR “CF liver”[tw] OR "CF lung"[tw] OR “CF pancreas”[tw] OR CF-related[tw] OR CFRD[tw])) AND ((("Hospice Care"[Mesh] OR "Hospice and Palliative Care Nursing"[Mesh] OR "Hospices"[Mesh] OR "Life Support Care"[Mesh:NoExp] OR "Palliative Care"[Mesh] OR "Palliative Medicine"[Mesh] OR "Terminal Care"[Mesh] OR "Terminally Ill"[Mesh] OR "Actively dying"[tw] OR "End of life"[All Fields] OR EOL[tiab] OR “goals of care”[tw] OR hospice*[tiab] OR hospice*[ot] OR “imminently dying”[tw] OR “life support”[tw] OR "Last year of life"[tiab] OR Palliative[tw] OR Palliative[ot] OR Palliative care[ot] OR “Pediatric end-of-life care”[ot] OR "place of death"[tw] OR “preferred place of care and death”[ot] OR "site of death"[tw] OR “symptom assessment”[tiab] OR “Symptom burden”[tw] OR “Symptom control”[ot] OR “symptom distress”[tw] OR “symptom management”[tw] OR "terminal care"[tw] OR "terminal illness"[tw] OR terminal patient*[tiab] OR "Terminal phase"[tw] OR "terminal stage"[tiab] OR "terminal stages"[tiab] OR “terminally ill”[tw])) OR ("Advance Care Planning"[Mesh] OR "Aftercare"[Mesh:NoExp] OR "Attitude to Death"[Mesh] OR "Bereavement"[Majr] OR "Caregivers"[MAJR] OR "Communication"[Majr:NoExp] OR "Critical Illness"[MAJR] OR "Depression"[Majr] OR "Disease Progression"[Mesh] OR "Dyspnea"[Mesh] OR "Fatigue"[Majr] OR "Interdisciplinary Communication"[Majr] OR "Life Expectancy"[Mesh] OR "Long-Term Care"[Majr] OR “Pain/drug therapy"[Majr:NoExp] OR "Pain/prevention and control"[Majr:NoExp] OR “Pain Management"[Majr] OR "Pain Measurement"[Majr] OR "Patient Care Team"[Mesh:NoExp] OR "Patient Comfort"[Mesh] OR "Patient Satisfaction"[Majr] OR "Prognosis"[Mesh:NoExp] OR "Quality of Life"[Mesh] OR "Respiration, Artificial"[Mesh] OR "Resuscitation Orders"[Mesh] OR "Spirituality"[Mesh] OR "Advance Care Planning"[ot] OR "Advance care planning"[tiab] OR "advance directives"[tw] OR "advanced directives"[tw] OR bereavement[ot] OR bereave*[title] OR Burden[ot] OR “Burden of treatment”[ot] OR “care coordination”[tiab] OR “care transitions”[tiab] OR “Caregiver burden”[ot] OR “comfort care”[tiab] OR “comforting measures”[ot] OR communication[ot] OR “critically ill”[tiab] OR “critical illness”[tiab] OR depress*[tiab] OR decision-making[ot] OR “disease burden”[tiab] OR “disease progression”[ot] OR “do not resuscitate”[tw] OR Dyspnea[tw] OR Dyspnoea[tw] OR "end stage"[tw] OR “family‐centered care”[ot] OR “family‐centred care”[ot] OR “family meetings”[ot] OR “Future Care Planning”[tiab] OR Interdisciplinary[tiab] OR “Information needs”[ot] OR “Interprofessional communication”[ot] OR interprofessional team*[ot] OR “Life expectancy”[ot] OR "Life-limiting"[tiab] OR Life-limiting[ot] OR life-threatening diagnos*[tiab] OR "life-threatening illness"[tiab] OR "limited survival"[tiab] OR “living will”[tw] OR “mechanical ventilation”[tw] OR Mortality[ot] OR Multidisciplinary[tiab] OR “Multidisciplinary management”[ot] OR “multidisciplinary team”[tiab] OR "Patient Satisfaction"[ot] OR "quality of life"[tw] OR QOL[tiab] OR Prognosis[tiab] OR Prognosis[ot] OR Prognostication[tiab] OR Prognostic disclosure*[tiab] OR Psychological[ot] OR Psychology[ot] OR Psychosocial[ot] OR “serious illness”[ot] OR “seriously ill”[tiab] OR spiritual*[tw] OR "supportive care"[tw] OR Survival[ot] OR symptom assessment[ot] OR Symptom burden[tw] OR “Symptom control”[tw] OR “symptom distress”[tw] OR “symptom management”[tiab] OR team[tiab] OR team-based care[ot] OR “treatment burden”[tw])))) OR ((("Cystic Fibrosis"[Mesh] OR "Bodily Secretions"[Mesh:NoExp] OR "Mucus/secretion"[Mesh:noexp] OR “Lung Transplantation”[MAJR] OR Cystic Fibrosis Foundation[Corporate Author] OR ECORN-CF Study Group[Corporate Author] OR ECORN-CF[All Fields] OR "European cystic fibrosis"[All Fields] OR “European Cystic Fibrosis Society”[Corporate Author] OR ECFS Board[Corporate Author] OR “European Cystic Fibrosis Society (ECFS) and the ECFS Clinical Trial Network”[Corporate Author] OR “European Cystic Fibrosis Society Clinical Trial Network Standardisation Committee”[Corporate Author] OR “International Committee on Mental Health”[Corporate Author] OR “ERS/ECFS Task Force on Provision of Care for Adults with Cystic Fibrosis in Europe”[Corporate Author] OR "Journal of cystic fibrosis : official journal of the European Cystic Fibrosis Society"[Jour] OR “US Cystic Fibrosis Foundation and European Cystic Fibrosis Society”[Corporate Author] OR "Cystic Fibrosis"[All Fields] OR “cystic lung disease”[tw] OR "CF Foundation"[tw] OR “CF liver”[tw] OR "CF lung"[tw] OR “CF pancreas”[tw] OR CF-related[tw] OR CFRD[tw] OR “CFTR-related”[tiab] OR Respiratory secretion[ot])) AND ("The American journal of hospice care"[Jour] OR "The American journal of hospice & palliative care"[Jour] OR "Annals of palliative medicine"[Jour] OR "BMC palliative care"[Jour] OR "BMJ supportive & palliative care"[Jour] OR "Current opinion in supportive and palliative care"[Jour] OR "Death Educ"[jour] OR "Death Stud."[jour] OR "The Hospice journal"[Jour] OR "Indian journal of palliative care"[Jour] OR "International journal of palliative nursing"[Jour] OR "Journal of hospice and palliative nursing : JHPN : the official journal of the Hospice and Palliative Nurses Association"[Jour] OR "Journal of geriatrics and palliative care"[Jour] OR "Journal of neuropathic pain & symptom palliation"[Jour] OR "Journal of pain & palliative care pharmacotherapy"[Jour] OR "Journal of pain and symptom management"[Jour] OR "Journal of palliative care"[Jour] OR "Journal of palliative medicine"[Jour] OR "Journal of social work in end-of-life & palliative care"[Jour] OR "Omega (Westport)."[jour] OR "Palliative & supportive care"[Jour] OR "J Support Oncol"[jour] OR "Palliative care"[Jour] OR "Palliative medicine"[Jour] OR "Progress in palliative care"[Jour] OR "Supportive care in cancer : official journal of the Multinational Association of Supportive Care in Cancer"[Jour]))) OR (CFQoL[tw] OR “QUAL-E”[ti] OR "CF-CARES"[tw] OR CF-MBQ[tiab] OR “Chronic Respiratory Questionnaire”[tiab] OR CF-MBQ[tiab] OR CF-SPS[tw] OR CFQoL[tw] OR CFQ-R[tw] OR “Cystic Fibrosis Questionnaire”[tw] OR “Cystic Fibrosis Questionnaire-Revised”[tiab] OR “Cystic Fibrosis Respiratory Symptom Diary”[tw] OR CFRSD[tiab] OR “FoP questionnaire”[tiab] OR “ProPal-COPD”[tw] OR ReS-CF[tiab] OR “STARx Questionnaire”[tiab] OR “STARx-P Questionnaire”[tiab])) OR (("Palliative Care"[MAJR] OR "Hospice and Palliative Care Nursing"[MAJR] OR "Palliative Medicine"[MAJR] OR "Palliative Care"[ti]) AND (instruments[ti] OR measures[ti] OR "QUAL-E"[tiab] OR scale*[ti] OR score*[ti] OR toolkit*[ti] OR tools[ti]))) OR (("generalist palliative care"[tw] OR “primary palliative care”[tw] OR palliative care curricula [All Fields] OR palliative care curriculum[All Fields] OR "SECTION ON HOSPICE AND PALLIATIVE MEDICINE"[Corporate Author]))) OR ((("Hospice and Palliative Care Nursing/education"[Mesh] OR "Palliative Medicine/education"[Mesh] OR "Palliative Care/manpower"[Mesh] OR “Palliative Care/organization and administration"[Mesh] OR “Palliative Care/standards"[Mesh] OR "palliative care clinicians"[tw] OR "palliative care clinician"[tw] OR hospice clinician*[tw] OR “integrated palliative care”[tiab] OR “palliative care service”[tiab] OR palliative care specialist*[tiab] OR “specialised palliative care”[tiab] OR “specialist palliative care”[tiab] OR “specialized palliative care”[tiab]) AND ("Attitude of Health Personnel"[MAJR:NoExp] OR "Clinical Competence"[Mesh] OR "Competency-Based Education"[Mesh] OR "Computer-Assisted Instruction"[MAJR] OR "Curriculum"[Mesh] OR "Education"[Majr:NoExp] OR “education”[Subheading] OR "Education, Distance"[MAJR] OR "Education, Professional"[Mesh] OR "Educational Measurement"[Mesh] OR "Educational Personnel"[MAJR] OR "Educational Status"[Majr] OR "Educational Technology"[Majr] OR "Health Knowledge, Attitudes, Practice"[Majr] OR "Health Occupations/education"[Mesh] OR "Health Personnel/education"[Mesh] OR "Learning"[Majr] OR "Models, Educational"[Mesh] OR "Needs Assessment"[Mesh] OR "Professional Competence"[Mesh] OR "Schools, Health Occupations"[Majr] OR "Students, Health Occupations"[Majr] OR "Teaching"[Mesh] OR "Training Support"[Majr] OR Class[ti] OR “clinical competence”[tiab] OR Course[ti] OR “communication course”[tiab] OR “Course goals”[tiab] OR “Course objectives”[tiab] OR Coursework[tiab] OR “Course work”[tiab] OR Curricula[tw] OR curricular[tiab] OR curriculum[tw] OR “e-learning”[tiab] OR education[ot] OR “educational interventions”[ot] OR “entrustable professional activities”[tiab] OR Instruction*[ti] OR “instructional design”[tiab] OR “instructional techniques” [tiab] OR “Interprofessional education”[tiab] OR learning[ti] OR “Learning objectives”[tiab] OR” Medical educational”[tiab] OR “needs assessment”[tw] OR “Peer learning”[ot] OR Skills[tw] OR "Specialization"[MAJR] OR Student*[ti] OR Teaching[tiab] OR “Team Training”[ot] OR trainee*[ti] OR Training[tw])) AND (("Acquired Immunodeficiency Syndrome"[Majr] OR "Anemia, Sickle Cell"[Majr:NoExp] OR "Catastrophic Illness"[Mesh] OR "COPD, Severe Early-Onset" [Supplementary Concept] OR “cystic fibrosis”[All Fields] OR "Dyspnea"[Majr] OR "Heart Defects, Congenital"[Majr] OR "HIV Infections"[Majr] OR "Hypertension, Pulmonary"[Majr] OR "Intensive Care Units, Pediatric"[Majr] OR "Organ Transplantation"[Majr] OR "Parents/psychology"[Majr] OR "Pediatrics"[Majr:NoExp] OR "Pulmonary Disease, Chronic Obstructive"[Majr] OR "Pulmonary Fibrosis"[Majr] OR "Pulmonary Medicine"[Mesh] OR "Renal Insufficiency, Chronic"[Majr] OR "Respiratory Therapy"[Majr] OR "Transition to Adult Care"[MAJR] OR "advanced cancer" [ti] OR "advanced disease"[tiab] OR "advanced heart"[ti] OR "advanced illness"[tiab] OR "advanced liver"[ti] OR "advanced lung"[tiab] OR advanced stage*[ti] OR Breathlessness[tw] OR "Breathlessness service"[ot] OR outcomes[ti] OR "Chronic disease"[tiab] OR "chronic lung"[tw] OR "Chronic obstructive pulmonary disease"[ot] OR "Chronic obstructive pulmonary disease"[ti] OR COPD[ti] OR "End-stage renal disease"[tw] OR HIV[ot] OR "idiopathic pulmonary fibrosis"[ot] OR "lung disease"[ot] OR “lung neoplasms”[ot] OR "mechanical ventilation"[tw] OR Oncology[ti] OR Paediatric[ti] OR "paediatric nursing"[ot] OR "Paediatric palliative care"[tiab] OR Pediatric*[ot] OR "pediatric end of life"[tiab] OR pediatric hematology/oncology[ot] OR "pediatric intensive care"[ot] OR "pediatric oncology"[tw] OR "pediatric palliative care"[tw] OR "progressive lung"[tiab] OR "Pulmonary arterial hypertension"[tw] OR "Pulmonary disease"[ot] OR Pulmonologist*[ot] OR "renal failure"[ti] OR Respiratory[ti] OR "serious illness"[ti] OR "seriously ill"[ti] OR "transition care"[ot] OR "transitional care"[tw] OR "transition-to-care"[tw] OR ”Transitions in Care”[ot] OR "transition of care"[tiab] OR transplant[ti] OR transplantation[ot] OR "advanced lung"[tiab] OR advanced respiratory[ot] OR "chronic lung"[tw] OR chronic respiratory[ti] OR "End-stage kidney disease"[tiab] OR "End-stage pulmonary disease"[ot] OR "end-stage renal disease"[ti] OR "Pulmonary rehabilitation"[ot] OR pulmonary re-transplantation[ot] OR pulmonologist*[tw] OR "Respiratory conditions"[tw] OR respiratory disease*[tw]) OR (“life limiting"[tw] AND respiratory[tw])))) OR (((palliative care model[tw] OR palliative care models[tw])) OR (("Models, Educational"[Mesh] OR "Models, Nursing"[MAJR] OR "Models, Organizational"[MAJR] OR Coaching[tiab] OR Model*[ti] OR Modeling[ot] OR “model of care”[tw] OR “models of care”[tw]) AND (Hospice[ti] OR hospices[ti] OR Palliative[ti] OR terminally[ti])))) OR ((((((("Hospice Care"[MAJR] OR "Hospice and Palliative Care Nursing"[MAJR] OR "Hospices"[MAJR] OR "Palliative Care"[Majr] OR "Palliative Medicine"[Mesh] OR "Terminal Care"[MAJR] OR "Terminally Ill"[MAJR] OR "Actively dying"[tw] OR "End of life"[All Fields] OR EOL[tiab] OR “goals of care”[tw] OR hospice*[ti] OR hospice*[ot] OR “imminently dying”[tw] OR "Last year of life"[ti] OR Palliative[ti] OR Palliative care[ot] OR Pediatric end-of-life care[ot] OR “symptom assessment”[ti] OR “Symptom burden”[tiab] OR “Symptom control”[ot] OR “symptom distress”[tiab] OR “symptom management”[tw] OR "terminal care"[tiab] OR "terminal illness"[tiab] OR terminal patient*[ti] OR “terminally ill”[tw])) AND ("Consensus"[Majr] OR "Consensus Development Conferences as Topic"[Majr] OR "Consensus Development Conference" [Publication Type] OR "Critical Pathways"[Mesh] OR “Evidence-Based Practice"[MAJR] OR "Guideline"[Publication Type] OR "Guideline Adherence"[MAJR] OR "Guidelines as Topic"[Mesh] OR "Meta-Analysis" [Publication Type] OR "Meta-Analysis as Topic"[Mesh] OR "Practice Guideline" [Publication Type] OR "Review"[pt] OR "Standard of Care"[Mesh] OR "Standing Orders"[Majr] OR "standards"[ti] OR “best practice”[tw] OR Consensus[tiab] OR “Critical pathways”[tw] OR Guideline[tw] OR Guidelines[ot] OR “Standards of care”[ot] OR “systematic review”[ti]))) AND ((((("Acquired Immunodeficiency Syndrome"[Majr] OR "Anemia, Sickle Cell"[Majr:NoExp] OR "Catastrophic Illness"[Mesh] OR "COPD, Severe Early-Onset" [Supplementary Concept] OR “cystic fibrosis”[All Fields] OR "Dyspnea"[Majr] OR "Heart Defects, Congenital"[Majr] OR "HIV Infections"[Majr] OR "Hypertension, Pulmonary"[Majr] OR "Intensive Care Units, Pediatric"[Majr] OR "Organ Transplantation"[Majr] OR "Pediatrics"[Majr:NoExp] OR "Pulmonary Disease, Chronic Obstructive"[Majr] OR "Pulmonary Fibrosis"[Majr] OR "Pulmonary Medicine"[Majr] OR "Renal Insufficiency, Chronic"[Majr] OR "Respiratory Therapy"[Majr] OR "Transition to Adult Care"[MAJR])) OR ("advanced cancer" [ti] OR "advanced disease"[ti] OR "advanced heart"[ti] OR "advanced illness"[ti] OR "advanced liver"[ti] OR "advanced lung"[tiab] OR “advanced stages”[ti] OR Breathlessness[tiab] OR "Breathlessness service"[ot] OR “caregiver outcomes”[ti] OR "chronic lung"[tw] OR "Chronic obstructive pulmonary disease"[ot] OR "Chronic obstructive pulmonary disease"[ti] OR COPD[ti] OR "End-stage renal disease"[ti] OR HIV[ot] OR "idiopathic pulmonary fibrosis"[ot] OR "lung disease"[ot] OR “lung neoplasms”[ot] OR "mechanical ventilation"[tiab] OR Oncology[ti] OR Paediatric[ti] OR "paediatric nursing"[ot] OR "Paediatric palliative care"[tiab] OR Pediatric*[ot] OR "pediatric end of life"[tiab] OR “pediatric hematology/oncology”[ot] OR "pediatric intensive care"[ot] OR "pediatric oncology"[ti] OR "pediatric palliative care"[tw] OR "progressive lung"[tiab] OR "Pulmonary arterial hypertension"[tw] OR "Pulmonary disease"[ot] OR Pulmonologist*[ot] OR "renal failure"[ti] OR Respiratory[ti] OR "serious illness"[ti] OR "seriously ill"[ti] OR "transitional care"[tw] OR “Transitions in Care”[ot] OR "transition of care"[tiab] OR transplant[ti] OR transplantation[ot])) OR ("advanced lung"[tiab] OR advanced respiratory[ot] OR "chronic lung"[tw] OR chronic respiratory[ti] OR "End-stage kidney disease"[tw] OR "End-stage pulmonary disease"[ot] OR "end-stage renal disease"[ti] OR pulmonary re-transplantation[ot] OR pulmonologist*[tw] OR "Respiratory conditions"[tiab] OR respiratory disease*[ti])) OR (“life limiting"[tw] AND respiratory[tw])))))) OR ((((("Checklist"[Majr] OR "Forced Expiratory Volume"[Mesh] OR "Respiratory Function Tests"[Mesh] OR checklist[tiab] OR “FEV1”[tw] OR “FEV(1)”[ot] OR Leicester Cough Questionnaire*[tiab] OR LifeCourse[tiab] OR “London Chest Activities”[tiab] OR "Lung Clearance Index"[tw] OR "lung allocation score"[All Fields] OR “Respiratory Distress Observation Scale”[tiab] OR "respiratory symptom diary"[tiab] OR “Respiratory Symptom Questionnaire”[tw] OR “Saint George's Respiratory Questionnaire”[tiab] OR “St George's Respiratory Questionnaire”[tiab] OR SGRQ[tiab] OR” Sino-Nasal Outcome Test”[tw] OR “SN-5”[tw] OR SNOT-14[tw] OR SNOT-22[tw])) AND ("Hospice Care"[Mesh] OR "Hospice and Palliative Care Nursing"[Mesh] OR "Hospices"[Mesh] OR "Life Support Care"[Mesh:NoExp] OR "Palliative Care"[Mesh] OR "Palliative Medicine"[Mesh] OR "Terminal Care"[Mesh] OR "Terminally Ill"[Mesh] OR "Actively dying"[tw] OR "End of life"[All Fields] OR EOL[tiab] OR “goals of care”[tw] OR hospice*[tiab] OR hospice*[ot] OR “imminently dying”[tw] OR “life support”[tw] OR "Last year of life"[tiab] OR Palliative[tw] OR Palliative[ot] OR Palliative care[ot] OR Pediatric end-of-life care[ot] OR "place of death"[tw] OR “preferred place of care and death”[ot] OR "site of death"[tw] OR “symptom assessment”[tiab] OR “Symptom burden”[tw] OR “Symptom control”[ot] OR “symptom distress”[tw] OR “symptom management”[tw] OR "terminal care"[tw] OR "terminal illness"[tw]))) AND (("Acquired Immunodeficiency Syndrome"[Majr] OR "Anemia, Sickle Cell"[Majr:NoExp] OR "Catastrophic Illness"[Mesh] OR "COPD, Severe Early-Onset" [Supplementary Concept] OR “cystic fibrosis”[All Fields] OR "Dyspnea"[Majr] OR "Heart Defects, Congenital"[Majr] OR "HIV Infections"[Majr] OR "Hypertension, Pulmonary"[Majr] OR "Intensive Care Units, Pediatric"[Majr] OR "Organ Transplantation"[Majr] OR "Parents/psychology"[Majr] OR "Pediatrics"[Majr:NoExp] OR "Pulmonary Disease, Chronic Obstructive"[Majr] OR "Pulmonary Fibrosis"[Majr] OR "Pulmonary Medicine"[Mesh] OR "Renal Insufficiency, Chronic"[Majr] OR "Respiratory Therapy"[Majr] OR "Transition to Adult Care"[MAJR] OR "advanced cancer" [ti] OR "advanced cancer"[ot] OR "advanced disease"[tiab] OR "advanced heart"[ot] OR "advanced illness"[tiab] OR "advanced liver"[tiab] OR "advanced lung"[tiab] OR “advanced stage”[ti] OR Breathlessness[tw] OR "Breathlessness service"[ot] OR "Chronic disease"[tw] OR "chronic lung"[tw] OR "Chronic obstructive pulmonary disease"[ot] OR "Chronic obstructive pulmonary disease"[ti] OR COPD[ti] OR "End-stage renal disease"[tw] OR HIV[ot] OR "idiopathic pulmonary fibrosis"[ot] OR Incurable[tiab] OR "lung disease"[ot] OR lung neoplasms[ot] OR "mechanical ventilation"[tw] OR Oncology[ti] OR Paediatric[ti] OR "paediatric nursing"[ot] OR "Paediatric palliative care"[tiab] OR Pediatric*[ot] OR "pediatric end of life"[tiab] OR pediatric hematology/oncology[ot] OR "pediatric intensive care"[ot] OR "pediatric oncology"[tw] OR "pediatric palliative care"[tw] OR "progressive lung"[tiab] OR "Pulmonary arterial hypertension"[tw] OR "Pulmonary disease"[ot] OR Pulmonologist*[ot] OR "renal failure"[tw] OR Respiratory[ti] OR "serious illness"[ti] OR "seriously ill"[ti] OR "transition care"[ot] OR "transitional care"[tw] OR "transition-to-care"[tw] OR “Transitions in Care”[ot] OR "transition of care"[tiab] OR transplant[ti] OR transplantation[ot] OR "advanced lung"[tiab] OR advanced respiratory[ot] OR "chronic lung"[tw] OR chronic respiratory[ti] OR "End-stage kidney disease"[tw] OR "End-stage pulmonary disease"[ot] OR "end-stage renal disease"[ti] OR "Pulmonary rehabilitation"[ot] OR pulmonary re-transplantation[ot] OR pulmonologist*[tw] OR "Respiratory conditions"[tw] OR respiratory disease*[tw]) OR (“life limiting"[tw] AND respiratory[tw])))) AND English[lang] AND "Humans"[Mesh])) OR ((((((((((((((("Cystic Fibrosis"[Mesh] OR Cystic Fibrosis Foundation[Corporate Author] OR ECORN-CF Study Group[Corporate Author] OR ECORN-CF[All Fields] OR "European cystic fibrosis"[All Fields] OR “European Cystic Fibrosis Society”[Corporate Author] OR ECFS Board[Corporate Author] OR “European Cystic Fibrosis Society (ECFS) and the ECFS Clinical Trial Network”[Corporate Author] OR “European Cystic Fibrosis Society Clinical Trial Network Standardisation Committee”[Corporate Author] OR “International Committee on Mental Health”[Corporate Author] OR “ERS/ECFS Task Force on Provision of Care for Adults with Cystic Fibrosis in Europe”[Corporate Author] OR "Journal of cystic fibrosis : official journal of the European Cystic Fibrosis Society"[Jour] OR “US Cystic Fibrosis Foundation and European Cystic Fibrosis Society”[Corporate Author] OR "Cystic Fibrosis"[All Fields] OR “cystic lung disease”[tw] OR "CF Foundation"[tw] OR “CF liver”[tw] OR "CF lung"[tw] OR “CF pancreas”[tw] OR CF-related[tw] OR CFRD[tw])) AND ((("Hospice Care"[Mesh] OR "Hospice and Palliative Care Nursing"[Mesh] OR "Hospices"[Mesh] OR "Life Support Care"[Mesh:NoExp] OR "Palliative Care"[Mesh] OR "Palliative Medicine"[Mesh] OR "Terminal Care"[Mesh] OR "Terminally Ill"[Mesh] OR "Actively dying"[tw] OR "End of life"[All Fields] OR EOL[tiab] OR “goals of care”[tw] OR hospice*[tiab] OR hospice*[ot] OR “imminently dying”[tw] OR “life support”[tw] OR "Last year of life"[tiab] OR Palliative[tw] OR Palliative[ot] OR Palliative care[ot] OR “Pediatric end-of-life care”[ot] OR "place of death"[tw] OR “preferred place of care and death”[ot] OR "site of death"[tw] OR “symptom assessment”[tiab] OR “Symptom burden”[tw] OR “Symptom control”[ot] OR “symptom distress”[tw] OR “symptom management”[tw] OR "terminal care"[tw] OR "terminal illness"[tw] OR terminal patient*[tiab] OR "Terminal phase"[tw] OR "terminal stage"[tiab] OR "terminal stages"[tiab] OR “terminally ill”[tw])) OR ("Advance Care Planning"[Mesh] OR "Aftercare"[Mesh:NoExp] OR "Attitude to Death"[Mesh] OR "Bereavement"[Majr] OR "Caregivers"[MAJR] OR "Communication"[Majr:NoExp] OR "Critical Illness"[MAJR] OR "Depression"[Majr] OR "Disease Progression"[Mesh] OR "Dyspnea"[Mesh] OR "Fatigue"[Majr] OR "Interdisciplinary Communication"[Majr] OR "Life Expectancy"[Mesh] OR "Long-Term Care"[Majr] OR “Pain/drug therapy"[Majr:NoExp] OR "Pain/prevention and control"[Majr:NoExp] OR “Pain Management"[Majr] OR "Pain Measurement"[Majr] OR "Patient Care Team"[Mesh:NoExp] OR "Patient Comfort"[Mesh] OR "Patient Satisfaction"[Majr] OR "Prognosis"[Mesh:NoExp] OR "Quality of Life"[Mesh] OR "Respiration, Artificial"[Mesh] OR "Resuscitation Orders"[Mesh] OR "Spirituality"[Mesh] OR "Advance Care Planning"[ot] OR "Advance care planning"[tiab] OR "advance directives"[tw] OR "advanced directives"[tw] OR bereavement[ot] OR bereave*[title] OR Burden[ot] OR “Burden of treatment”[ot] OR “care coordination”[tiab] OR “care transitions”[tiab] OR “Caregiver burden”[ot] OR “comfort care”[tiab] OR “comforting measures”[ot] OR communication[ot] OR “critically ill”[tiab] OR “critical illness”[tiab] OR depress*[tiab] OR decision-making[ot] OR “disease burden”[tiab] OR “disease progression”[ot] OR “do not resuscitate”[tw] OR Dyspnea[tw] OR Dyspnoea[tw] OR "end stage"[tw] OR “family‐centered care”[ot] OR “family‐centred care”[ot] OR “family meetings”[ot] OR “Future Care Planning”[tiab] OR Interdisciplinary[tiab] OR “Information needs”[ot] OR “Interprofessional communication”[ot] OR interprofessional team*[ot] OR “Life expectancy”[ot] OR "Life-limiting"[tiab] OR Life-limiting[ot] OR life-threatening diagnos*[tiab] OR "life-threatening illness"[tiab] OR "limited survival"[tiab] OR “living will”[tw] OR “mechanical ventilation”[tw] OR Mortality[ot] OR Multidisciplinary[tiab] OR “Multidisciplinary management”[ot] OR “multidisciplinary team”[tiab] OR "Patient Satisfaction"[ot] OR "quality of life"[tw] OR QOL[tiab] OR Prognosis[tiab] OR Prognosis[ot] OR Prognostication[tiab] OR Prognostic disclosure*[tiab] OR Psychological[ot] OR Psychology[ot] OR Psychosocial[ot] OR “serious illness”[ot] OR “seriously ill”[tiab] OR spiritual*[tw] OR "supportive care"[tw] OR Survival[ot] OR symptom assessment[ot] OR Symptom burden[tw] OR “Symptom control”[tw] OR “symptom distress”[tw] OR “symptom management”[tiab] OR team[tiab] OR team-based care[ot] OR “treatment burden”[tw])))) OR ((("Cystic Fibrosis"[Mesh] OR "Bodily Secretions"[Mesh:NoExp] OR "Mucus/secretion"[Mesh:noexp] OR “Lung Transplantation”[MAJR] OR Cystic Fibrosis Foundation[Corporate Author] OR ECORN-CF Study Group[Corporate Author] OR ECORN-CF[All Fields] OR "European cystic fibrosis"[All Fields] OR “European Cystic Fibrosis Society”[Corporate Author] OR ECFS Board[Corporate Author] OR “European Cystic Fibrosis Society (ECFS) and the ECFS Clinical Trial Network”[Corporate Author] OR “European Cystic Fibrosis Society Clinical Trial Network Standardisation Committee”[Corporate Author] OR “International Committee on Mental Health”[Corporate Author] OR “ERS/ECFS Task Force on Provision of Care for Adults with Cystic Fibrosis in Europe”[Corporate Author] OR "Journal of cystic fibrosis : official journal of the European Cystic Fibrosis Society"[Jour] OR “US Cystic Fibrosis Foundation and European Cystic Fibrosis Society”[Corporate Author] OR "Cystic Fibrosis"[All Fields] OR “cystic lung disease”[tw] OR "CF Foundation"[tw] OR “CF liver”[tw] OR "CF lung"[tw] OR “CF pancreas”[tw] OR CF-related[tw] OR CFRD[tw] OR “CFTR-related”[tiab] OR Respiratory secretion[ot])) AND ("The American journal of hospice care"[Jour] OR "The American journal of hospice & palliative care"[Jour] OR "Annals of palliative medicine"[Jour] OR "BMC palliative care"[Jour] OR "BMJ supportive & palliative care"[Jour] OR "Current opinion in supportive and palliative care"[Jour] OR "Death Educ"[jour] OR "Death Stud."[jour] OR "The Hospice journal"[Jour] OR "Indian journal of palliative care"[Jour] OR "International journal of palliative nursing"[Jour] OR "Journal of hospice and palliative nursing : JHPN : the official journal of the Hospice and Palliative Nurses Association"[Jour] OR "Journal of geriatrics and palliative care"[Jour] OR "Journal of neuropathic pain & symptom palliation"[Jour] OR "Journal of pain & palliative care pharmacotherapy"[Jour] OR "Journal of pain and symptom management"[Jour] OR "Journal of palliative care"[Jour] OR "Journal of palliative medicine"[Jour] OR "Journal of social work in end-of-life & palliative care"[Jour] OR "Omega (Westport)."[jour] OR "Palliative & supportive care"[Jour] OR "J Support Oncol"[jour] OR "Palliative care"[Jour] OR "Palliative medicine"[Jour] OR "Progress in palliative care"[Jour] OR "Supportive care in cancer : official journal of the Multinational Association of Supportive Care in Cancer"[Jour]))) OR (CFQoL[tw] OR “QUAL-E”[ti] OR "CF-CARES"[tw] OR CF-MBQ[tiab] OR “Chronic Respiratory Questionnaire”[tiab] OR CF-MBQ[tiab] OR CF-SPS[tw] OR CFQoL[tw] OR CFQ-R[tw] OR “Cystic Fibrosis Questionnaire”[tw] OR “Cystic Fibrosis Questionnaire-Revised”[tiab] OR “Cystic Fibrosis Respiratory Symptom Diary”[tw] OR CFRSD[tiab] OR “FoP questionnaire”[tiab] OR “ProPal-COPD”[tw] OR ReS-CF[tiab] OR “STARx Questionnaire”[tiab] OR “STARx-P Questionnaire”[tiab])) OR (("Palliative Care"[MAJR] OR "Hospice and Palliative Care Nursing"[MAJR] OR "Palliative Medicine"[MAJR] OR "Palliative Care"[ti]) AND (instruments[ti] OR measures[ti] OR "QUAL-E"[tiab] OR scale*[ti] OR score*[ti] OR toolkit*[ti] OR tools[ti]))) OR (("generalist palliative care"[tw] OR “primary palliative care”[tw] OR palliative care curricula [All Fields] OR palliative care curriculum[All Fields] OR "SECTION ON HOSPICE AND PALLIATIVE MEDICINE"[Corporate Author]))) OR ((("Hospice and Palliative Care Nursing/education"[Mesh] OR "Palliative Medicine/education"[Mesh] OR "Palliative Care/manpower"[Mesh] OR “Palliative Care/organization and administration"[Mesh] OR “Palliative Care/standards"[Mesh] OR "palliative care clinicians"[tw] OR "palliative care clinician"[tw] OR hospice clinician*[tw] OR “integrated palliative care”[tiab] OR “palliative care service”[tiab] OR palliative care specialist*[tiab] OR “specialised palliative care”[tiab] OR “specialist palliative care”[tiab] OR “specialized palliative care”[tiab]) AND ("Attitude of Health Personnel"[MAJR:NoExp] OR "Clinical Competence"[Mesh] OR "Competency-Based Education"[Mesh] OR "Computer-Assisted Instruction"[MAJR] OR "Curriculum"[Mesh] OR "Education"[Majr:NoExp] OR “education”[Subheading] OR "Education, Distance"[MAJR] OR "Education, Professional"[Mesh] OR "Educational Measurement"[Mesh] OR "Educational Personnel"[MAJR] OR "Educational Status"[Majr] OR "Educational Technology"[Majr] OR "Health Knowledge, Attitudes, Practice"[Majr] OR "Health Occupations/education"[Mesh] OR "Health Personnel/education"[Mesh] OR "Learning"[Majr] OR "Models, Educational"[Mesh] OR "Needs Assessment"[Mesh] OR "Professional Competence"[Mesh] OR "Schools, Health Occupations"[Majr] OR "Students, Health Occupations"[Majr] OR "Teaching"[Mesh] OR "Training Support"[Majr] OR Class[ti] OR “clinical competence”[tiab] OR Course[ti] OR “communication course”[tiab] OR “Course goals”[tiab] OR “Course objectives”[tiab] OR Coursework[tiab] OR “Course work”[tiab] OR Curricula[tw] OR curricular[tiab] OR curriculum[tw] OR “e-learning”[tiab] OR education[ot] OR “educational interventions”[ot] OR “entrustable professional activities”[tiab] OR Instruction*[ti] OR “instructional design”[tiab] OR “instructional techniques” [tiab] OR “Interprofessional education”[tiab] OR learning[ti] OR “Learning objectives”[tiab] OR” Medical educational”[tiab] OR “needs assessment”[tw] OR “Peer learning”[ot] OR Skills[tw] OR "Specialization"[MAJR] OR Student*[ti] OR Teaching[tiab] OR “Team Training”[ot] OR trainee*[ti] OR Training[tw])) AND (("Acquired Immunodeficiency Syndrome"[Majr] OR "Anemia, Sickle Cell"[Majr:NoExp] OR "Catastrophic Illness"[Mesh] OR "COPD, Severe Early-Onset" [Supplementary Concept] OR “cystic fibrosis”[All Fields] OR "Dyspnea"[Majr] OR "Heart Defects, Congenital"[Majr] OR "HIV Infections"[Majr] OR "Hypertension, Pulmonary"[Majr] OR "Intensive Care Units, Pediatric"[Majr] OR "Organ Transplantation"[Majr] OR "Parents/psychology"[Majr] OR "Pediatrics"[Majr:NoExp] OR "Pulmonary Disease, Chronic Obstructive"[Majr] OR "Pulmonary Fibrosis"[Majr] OR "Pulmonary Medicine"[Mesh] OR "Renal Insufficiency, Chronic"[Majr] OR "Respiratory Therapy"[Majr] OR "Transition to Adult Care"[MAJR] OR "advanced cancer" [ti] OR "advanced disease"[tiab] OR "advanced heart"[ti] OR "advanced illness"[tiab] OR "advanced liver"[ti] OR "advanced lung"[tiab] OR advanced stage*[ti] OR Breathlessness[tw] OR "Breathlessness service"[ot] OR outcomes[ti] OR "Chronic disease"[tiab] OR "chronic lung"[tw] OR "Chronic obstructive pulmonary disease"[ot] OR "Chronic obstructive pulmonary disease"[ti] OR COPD[ti] OR "End-stage renal disease"[tw] OR HIV[ot] OR "idiopathic pulmonary fibrosis"[ot] OR "lung disease"[ot] OR “lung neoplasms”[ot] OR "mechanical ventilation"[tw] OR Oncology[ti] OR Paediatric[ti] OR "paediatric nursing"[ot] OR "Paediatric palliative care"[tiab] OR Pediatric*[ot] OR "pediatric end of life"[tiab] OR pediatric hematology/oncology[ot] OR "pediatric intensive care"[ot] OR "pediatric oncology"[tw] OR "pediatric palliative care"[tw] OR "progressive lung"[tiab] OR "Pulmonary arterial hypertension"[tw] OR "Pulmonary disease"[ot] OR Pulmonologist*[ot] OR "renal failure"[ti] OR Respiratory[ti] OR "serious illness"[ti] OR "seriously ill"[ti] OR "transition care"[ot] OR "transitional care"[tw] OR "transition-to-care"[tw] OR ”Transitions in Care”[ot] OR "transition of care"[tiab] OR transplant[ti] OR transplantation[ot] OR "advanced lung"[tiab] OR advanced respiratory[ot] OR "chronic lung"[tw] OR chronic respiratory[ti] OR "End-stage kidney disease"[tiab] OR "End-stage pulmonary disease"[ot] OR "end-stage renal disease"[ti] OR "Pulmonary rehabilitation"[ot] OR pulmonary re-transplantation[ot] OR pulmonologist*[tw] OR "Respiratory conditions"[tw] OR respiratory disease*[tw]) OR (“life limiting"[tw] AND respiratory[tw])))) OR (((palliative care model[tw] OR palliative care models[tw])) OR (("Models, Educational"[Mesh] OR "Models, Nursing"[MAJR] OR "Models, Organizational"[MAJR] OR Coaching[tiab] OR Model*[ti] OR Modeling[ot] OR “model of care”[tw] OR “models of care”[tw]) AND (Hospice[ti] OR hospices[ti] OR Palliative[ti] OR terminally[ti])))) OR ((((((("Hospice Care"[MAJR] OR "Hospice and Palliative Care Nursing"[MAJR] OR "Hospices"[MAJR] OR "Palliative Care"[Majr] OR "Palliative Medicine"[Mesh] OR "Terminal Care"[MAJR] OR "Terminally Ill"[MAJR] OR "Actively dying"[tw] OR "End of life"[All Fields] OR EOL[tiab] OR “goals of care”[tw] OR hospice*[ti] OR hospice*[ot] OR “imminently dying”[tw] OR "Last year of life"[ti] OR Palliative[ti] OR Palliative care[ot] OR Pediatric end-of-life care[ot] OR “symptom assessment”[ti] OR “Symptom burden”[tiab] OR “Symptom control”[ot] OR “symptom distress”[tiab] OR “symptom management”[tw] OR "terminal care"[tiab] OR "terminal illness"[tiab] OR terminal patient*[ti] OR “terminally ill”[tw])) AND ("Consensus"[Majr] OR "Consensus Development Conferences as Topic"[Majr] OR "Consensus Development Conference" [Publication Type] OR "Critical Pathways"[Mesh] OR “Evidence-Based Practice"[MAJR] OR "Guideline"[Publication Type] OR "Guideline Adherence"[MAJR] OR "Guidelines as Topic"[Mesh] OR "Meta-Analysis" [Publication Type] OR "Meta-Analysis as Topic"[Mesh] OR "Practice Guideline" [Publication Type] OR "Review"[pt] OR "Standard of Care"[Mesh] OR "Standing Orders"[Majr] OR "standards"[ti] OR “best practice”[tw] OR Consensus[tiab] OR “Critical pathways”[tw] OR Guideline[tw] OR Guidelines[ot] OR “Standards of care”[ot] OR “systematic review”[ti]))) AND ((((("Acquired Immunodeficiency Syndrome"[Majr] OR "Anemia, Sickle Cell"[Majr:NoExp] OR "Catastrophic Illness"[Mesh] OR "COPD, Severe Early-Onset" [Supplementary Concept] OR “cystic fibrosis”[All Fields] OR "Dyspnea"[Majr] OR "Heart Defects, Congenital"[Majr] OR "HIV Infections"[Majr] OR "Hypertension, Pulmonary"[Majr] OR "Intensive Care Units, Pediatric"[Majr] OR "Organ Transplantation"[Majr] OR "Pediatrics"[Majr:NoExp] OR "Pulmonary Disease, Chronic Obstructive"[Majr] OR "Pulmonary Fibrosis"[Majr] OR "Pulmonary Medicine"[Majr] OR "Renal Insufficiency, Chronic"[Majr] OR "Respiratory Therapy"[Majr] OR "Transition to Adult Care"[MAJR])) OR ("advanced cancer" [ti] OR "advanced disease"[ti] OR "advanced heart"[ti] OR "advanced illness"[ti] OR "advanced liver"[ti] OR "advanced lung"[tiab] OR advanced stages*[ti] OR Breathlessness[tiab] OR "Breathlessness service"[ot] OR “caregiver outcomes”[ti] OR "chronic lung"[tw] OR "Chronic obstructive pulmonary disease"[ot] OR "Chronic obstructive pulmonary disease"[ti] OR COPD[ti] OR "End-stage renal disease"[ti] OR HIV[ot] OR "idiopathic pulmonary fibrosis"[ot] OR "lung disease"[ot] OR “lung neoplasms”[ot] OR "mechanical ventilation"[tiab] OR Oncology[ti] OR Paediatric[ti] OR "paediatric nursing"[ot] OR "Paediatric palliative care"[tiab] OR Pediatric*[ot] OR "pediatric end of life"[tiab] OR “pediatric hematology/oncology”[ot] OR "pediatric intensive care"[ot] OR "pediatric oncology"[ti] OR "pediatric palliative care"[tw] OR "progressive lung"[tiab] OR "Pulmonary arterial hypertension"[tw] OR "Pulmonary disease"[ot] OR Pulmonologist*[ot] OR "renal failure"[ti] OR Respiratory[ti] OR "serious illness"[ti] OR "seriously ill"[ti] OR "transitional care"[tw] OR “Transitions in Care”[ot] OR "transition of care"[tiab] OR transplant[ti] OR transplantation[ot])) OR ("advanced lung"[tiab] OR advanced respiratory[ot] OR "chronic lung"[tw] OR chronic respiratory[ti] OR "End-stage kidney disease"[tw] OR "End-stage pulmonary disease"[ot] OR "end-stage renal disease"[ti] OR pulmonary re-transplantation[ot] OR pulmonologist*[tw] OR "Respiratory conditions"[tiab] OR respiratory disease*[ti])) OR (“life limiting"[tw] AND respiratory[tw])))))) OR ((((("Checklist"[Majr] OR "Forced Expiratory Volume"[Mesh] OR "Respiratory Function Tests"[Mesh] OR checklist[tiab] OR “FEV1”[tw] OR “FEV(1)”[ot] OR Leicester Cough Questionnaire*[tiab] OR LifeCourse[tiab] OR “London Chest Activities”[tiab] OR "Lung Clearance Index"[tw] OR "lung allocation score"[All Fields] OR “Respiratory Distress Observation Scale”[tiab] OR "respiratory symptom diary"[tiab] OR “Respiratory Symptom Questionnaire”[tw] OR “Saint George's Respiratory Questionnaire”[tiab] OR “St George's Respiratory Questionnaire”[tiab] OR SGRQ[tiab] OR” Sino-Nasal Outcome Test”[tw] OR “SN-5”[tw] OR SNOT-14[tw] OR SNOT-22[tw])) AND ("Hospice Care"[Mesh] OR "Hospice and Palliative Care Nursing"[Mesh] OR "Hospices"[Mesh] OR "Life Support Care"[Mesh:NoExp] OR "Palliative Care"[Mesh] OR "Palliative Medicine"[Mesh] OR "Terminal Care"[Mesh] OR "Terminally Ill"[Mesh] OR "Actively dying"[tw] OR "End of life"[All Fields] OR EOL[tiab] OR “goals of care”[tw] OR hospice*[tiab] OR hospice*[ot] OR “imminently dying”[tw] OR “life support”[tw] OR "Last year of life"[tiab] OR Palliative[tw] OR Palliative[ot] OR Palliative care[ot] OR Pediatric end-of-life care[ot] OR "place of death"[tw] OR “preferred place of care and death”[ot] OR "site of death"[tw] OR “symptom assessment”[tiab] OR “Symptom burden”[tw] OR “Symptom control”[ot] OR “symptom distress”[tw] OR “symptom management”[tw] OR "terminal care"[tw] OR "terminal illness"[tw]))) AND (("Acquired Immunodeficiency Syndrome"[Majr] OR "Anemia, Sickle Cell"[Majr:NoExp] OR "Catastrophic Illness"[Mesh] OR "COPD, Severe Early-Onset" [Supplementary Concept] OR “cystic fibrosis”[All Fields] OR "Dyspnea"[Majr] OR "Heart Defects, Congenital"[Majr] OR "HIV Infections"[Majr] OR "Hypertension, Pulmonary"[Majr] OR "Intensive Care Units, Pediatric"[Majr] OR "Organ Transplantation"[Majr] OR "Parents/psychology"[Majr] OR "Pediatrics"[Majr:NoExp] OR "Pulmonary Disease, Chronic Obstructive"[Majr] OR "Pulmonary Fibrosis"[Majr] OR "Pulmonary Medicine"[Mesh] OR "Renal Insufficiency, Chronic"[Majr] OR "Respiratory Therapy"[Majr] OR "Transition to Adult Care"[MAJR] OR "advanced cancer" [ti] OR "advanced cancer"[ot] OR "advanced disease"[tiab] OR "advanced heart"[ot] OR "advanced illness"[tiab] OR "advanced liver"[tiab] OR "advanced lung"[tiab] OR advanced stage*[ti] OR Breathlessness[tw] OR "Breathlessness service"[ot] OR "Chronic disease"[tw] OR "chronic lung"[tw] OR "Chronic obstructive pulmonary disease"[ot] OR "Chronic obstructive pulmonary disease"[ti] OR COPD[ti] OR "End-stage renal disease"[tw] OR HIV[ot] OR "idiopathic pulmonary fibrosis"[ot] OR Incurable[tiab] OR "lung disease"[ot] OR lung neoplasms[ot] OR "mechanical ventilation"[tw] OR Oncology[ti] OR Paediatric[ti] OR "paediatric nursing"[ot] OR "Paediatric palliative care"[tiab] OR Pediatric*[ot] OR "pediatric end of life"[tiab] OR pediatric hematology/oncology[ot] OR "pediatric intensive care"[ot] OR "pediatric oncology"[tw] OR "pediatric palliative care"[tw] OR "progressive lung"[tiab] OR "Pulmonary arterial hypertension"[tw] OR "Pulmonary disease"[ot] OR Pulmonologist*[ot] OR "renal failure"[tw] OR Respiratory[ti] OR "serious illness"[ti] OR "seriously ill"[ti] OR "transition care"[ot] OR "transitional care"[tw] OR "transition-to-care"[tw] OR “Transitions in Care”[ot] OR "transition of care"[tiab] OR transplant[ti] OR transplantation[ot] OR "advanced lung"[tiab] OR advanced respiratory[ot] OR "chronic lung"[tw] OR chronic respiratory[ti] OR "End-stage kidney disease"[tw] OR "End-stage pulmonary disease"[ot] OR "end-stage renal disease"[ti] OR "Pulmonary rehabilitation"[ot] OR pulmonary re-transplantation[ot] OR pulmonologist*[tw] OR "Respiratory conditions"[tw] OR respiratory disease*[tw]) OR (“life limiting"[tw] AND respiratory[tw])))) AND English[lang])) NOT ("Animals"[Mesh:NoExp] OR "Animal Diseases"[Mesh] OR "Animal Population Groups"[Mesh] OR "Carnivora"[Mesh] OR "Disease Models, Animal"[Mesh] OR "Plasmids"[Mesh] OR "Rodentia"[Mesh] OR “animal model”[tw] OR “animal models”[tw] OR ferrets[tw] OR mice[tw] OR “mice model”[tw] OR “mice models”[tw] OR mouse[tw] OR murine[tw] OR plasmids[tw] OR rat[tw] OR rats[tw])))) NOT (“non cystic fibrosis” [All Fields] OR “noncystic fibrosis” [All Fields] OR "Cells, Cultured"[Mesh] OR "Depression, Chemical"[Mesh] OR "Swine"[Mesh] OR animal*[ti] OR animal model*[tiab] OR mice[ti] OR mouse[ti] OR pigs[ti] OR rat[ti] OR rats[ti] OR “Tertiary alcohols”[ot])

**3. Flow diagram of studies identified and reviewed to support the development of recommendation statements based on the Preferred Reporting of Items for Systematic Review and Meta-Analysis^1^.**


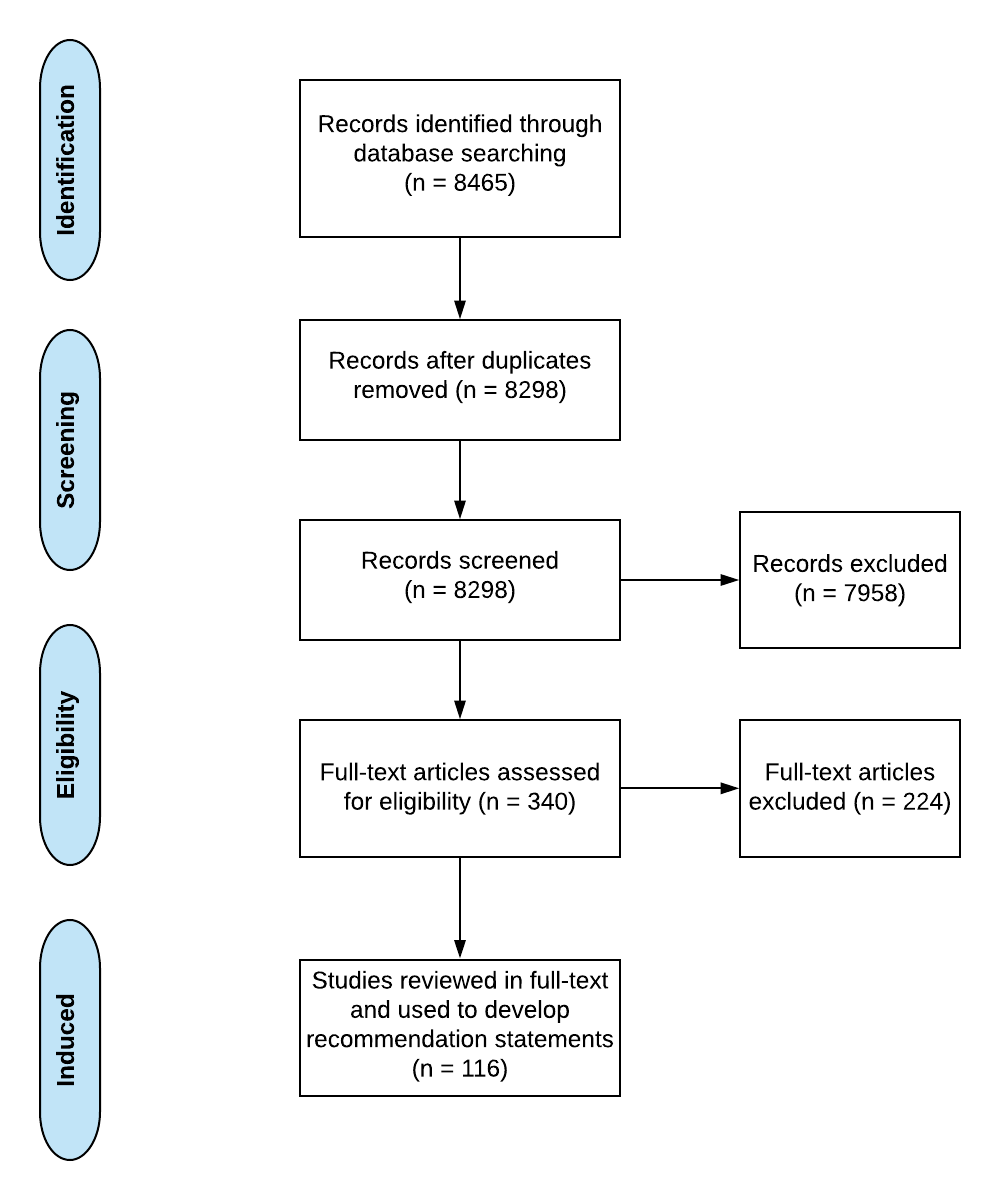


**4. Flow diagram of palliative care needs assessment tools identified by Workgroup 3 based on the Preferred Reporting of Items for Systematic Review and Meta-Analysis^1^.**


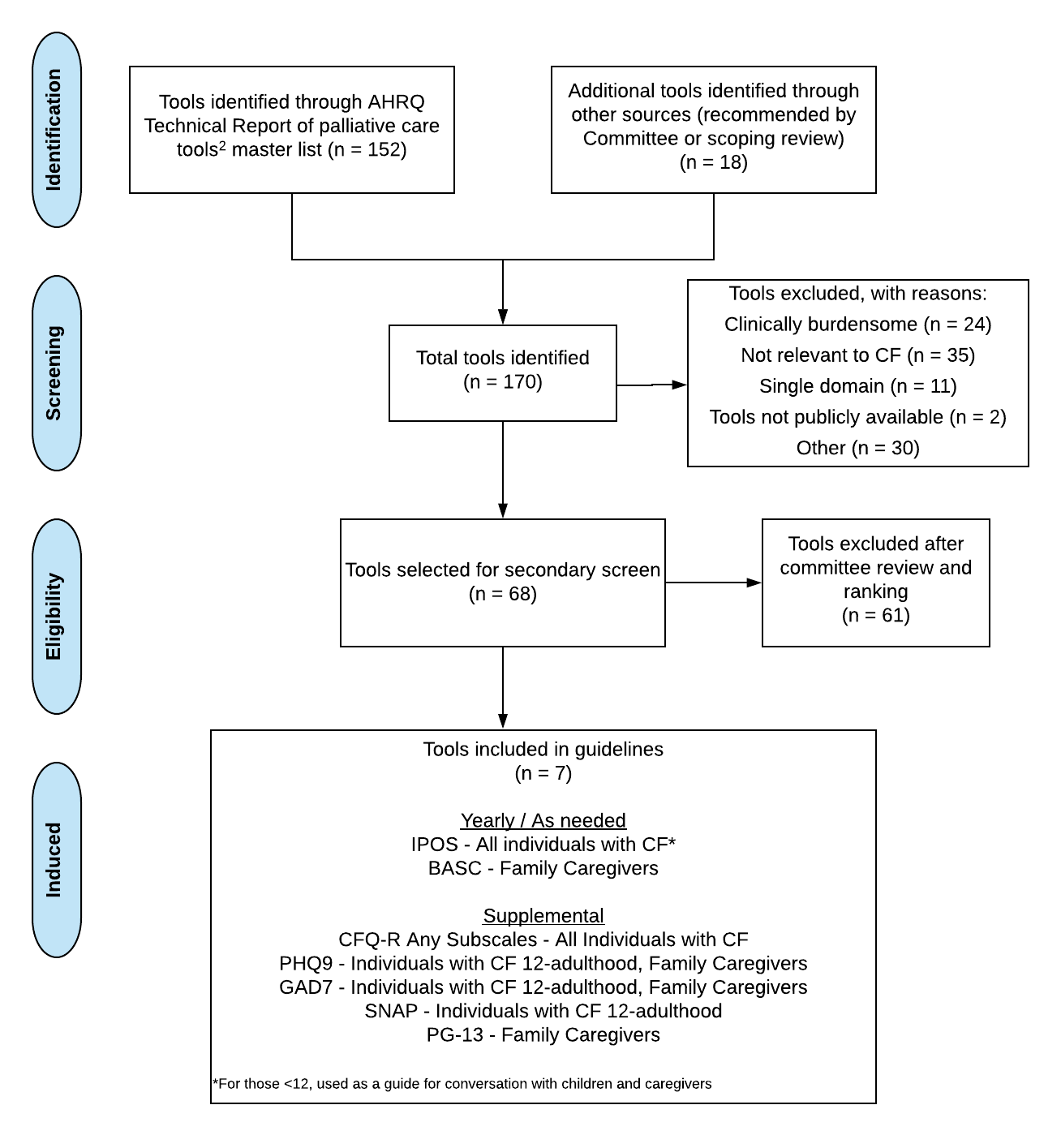


**5. Screening and Assessment Workgroup review strategy**

The screening and assessment workgroup aimed to develop guidelines to facilitate screening and clinical assessment by care team members in individuals with CF and family caregivers to identify palliative care needs annually and when major events trigger re-screening throughout the illness course. Screening for common concerns (e.g., physical/psychological symptoms, spiritual distress, and caregiver burden) is followed by clinical assessment to lead to an individualized palliative plan of care for management by the care team or other specialists. The approach used by the workgroup involved evaluating generic, multidimensional measures assessing multi-faceted aspects of health status (core), with the supplemental use of single domain measures (module) to allow for in-depth investigation of specific areas that individuals with CF identify as problematic.

Evaluation by workgroup members involved a consensus-based approach. The process is summarized here:

- Sources of measures obtained for evaluation: All 152 measures identified by Aslakson et al^2^ were reviewed for possible inclusion. We identified an additional 18 tools through other sources (Recommended by Committee or Scoping Review). The selection framework was: 1) generic health status measures for adults, 2) generic health status measures for children, 3) CF-specific measures for adults, 4) CF-specific measures for children, 5) non-CF specific, non-generics for adults and 6) non-CF specific, non-generics for children (See Supplementary Appendix, Table 1, Section 3.1).
- Criteria for measure evaluation: All measures were reviewed for initial inclusion criteria by at least 2 workgroup members. Measures meeting initial criteria were further reviewed and then ranked by at least 3 workgroup members (see below).
  - *Initial inclusion criteria*: 1) validated measures; 2) self-report measures; 3) relevant to CF-specific clinical needs (e.g., high frequency symptoms and concerns); 4) relevant for all stages of CF disease severity, including the end of life, 5) developmentally appropriate for children, adolescents, or adults, and 6) brief and non-burdensome for clinical use. *Exclusion criteria*: 1) Non-validated measures; 2) structured interviews (unless pediatric-focused); 3) not relevant to CF (e.g., specific to non-CF medical conditions), and 4) clinically-burdensome.
  - *Secondary inclusion criteria*: Measures meeting initial criteria were reviewed more extensively to identify those appropriate for ranking/final selection: 1) CF-specific; 2) non-CF specific measure previously used in CF populations; 3) non-CF specific measure previously used in non-CF, palliative care populations; 4) indicate clinically-actionable areas for care team intervention; 5) assess both spirituality and religiosity with cross-cultural applicability (spiritual measures); and 6) appropriate for caregivers of individuals with CF of all ages (caregiver measures).
- Eligible measures were then ranked (from “1-5”) and those receiving the top rating of “1” (see manuscript, Table 4) were approved for final selection by all workgroup members. Results revealed few available measures relevant to CF-specific clinical needs addressing several (sub)domains: structure and process, ethical and legal, culture, and patient-reported experience.

**REFERENCES FOR SUPPLEMENTARY APPENDIX**

1. Moher D, Liberati A, Tetzlaff J, Altman DG, Group P. Preferred reporting items for systematic reviews and meta-analyses: the PRISMA statement. In:2010.

2. Aslakson R, Dy SM, Wilson RF, et al. AHRQ Comparative Effectiveness Technical Briefs. *Assessment Tools for Palliative Care.* 2017.
